# Cross-species recognition and molecular basis of SARS-CoV-2 and SARS-CoV binding to ACE2s of marine animals

**DOI:** 10.1093/nsr/nwac122

**Published:** 2022-06-23

**Authors:** Shihua Li, Ruirui Yang, Di Zhang, Pu Han, Zepeng Xu, Qian Chen, Runchu Zhao, Xin Zhao, Xiao Qu, Anqi Zheng, Liang Wang, Linjie Li, Yu Hu, Rong Zhang, Chao Su, Sheng Niu, Yanfang Zhang, Jianxun Qi, Kefang Liu, Qihui Wang, George F Gao

**Affiliations:** CAS Key Laboratory of Pathogenic Microbiology and Immunology, Institute of Microbiology, Chinese Academy of Sciences, Beijing100101, China; CAS Key Laboratory of Pathogenic Microbiology and Immunology, Institute of Microbiology, Chinese Academy of Sciences, Beijing100101, China; College of Veterinary Medicine, Shanxi Agricultural University, Jinzhong030801, China; CAS Key Laboratory of Pathogenic Microbiology and Immunology, Institute of Microbiology, Chinese Academy of Sciences, Beijing100101, China; Faculty of Health Sciences, University of Macau, Macau, China; CAS Key Laboratory of Pathogenic Microbiology and Immunology, Institute of Microbiology, Chinese Academy of Sciences, Beijing100101, China; CAS Key Laboratory of Pathogenic Microbiology and Immunology, Institute of Microbiology, Chinese Academy of Sciences, Beijing100101, China; Faculty of Health Sciences, University of Macau, Macau, China; CAS Key Laboratory of Pathogenic Microbiology and Immunology, Institute of Microbiology, Chinese Academy of Sciences, Beijing100101, China; Institute of Physical Science and Information, Anhui University, Hefei230039, China; CAS Key Laboratory of Pathogenic Microbiology and Immunology, Institute of Microbiology, Chinese Academy of Sciences, Beijing100101, China; Institute of Physical Science and Information, Anhui University, Hefei230039, China; CAS Key Laboratory of Pathogenic Microbiology and Immunology, Institute of Microbiology, Chinese Academy of Sciences, Beijing100101, China; Center for Influenza Research and Early-Warning (CASCIRE), Chinese Academy of Sciences, Beijing100101, China; CAS Key Laboratory of Pathogenic Microbiology and Immunology, Institute of Microbiology, Chinese Academy of Sciences, Beijing100101, China; CAS Key Laboratory of Pathogenic Microbiology and Immunology, Institute of Microbiology, Chinese Academy of Sciences, Beijing100101, China; CAS Key Laboratory of Pathogenic Microbiology and Immunology, Institute of Microbiology, Chinese Academy of Sciences, Beijing100101, China; Center for Influenza Research and Early-Warning (CASCIRE), Chinese Academy of Sciences, Beijing100101, China; CAS Key Laboratory of Pathogenic Microbiology and Immunology, Institute of Microbiology, Chinese Academy of Sciences, Beijing100101, China; Savaid Medical School, University of Chinese Academy of Sciences, Beijing100049, China; CAS Key Laboratory of Pathogenic Microbiology and Immunology, Institute of Microbiology, Chinese Academy of Sciences, Beijing100101, China; School of Life Sciences, University of Science and Technology of China, Hefei230026, China; CAS Key Laboratory of Pathogenic Microbiology and Immunology, Institute of Microbiology, Chinese Academy of Sciences, Beijing100101, China; State Key Laboratory for Conservation and Utilization of Subtropical Agro-Bioresources, Guangxi University, Nanning530004, China; CAS Key Laboratory of Pathogenic Microbiology and Immunology, Institute of Microbiology, Chinese Academy of Sciences, Beijing100101, China; CAS Key Laboratory of Pathogenic Microbiology and Immunology, Institute of Microbiology, Chinese Academy of Sciences, Beijing100101, China; College of Veterinary Medicine, Shanxi Agricultural University, Jinzhong030801, China; CAS Key Laboratory of Pathogenic Microbiology and Immunology, Institute of Microbiology, Chinese Academy of Sciences, Beijing100101, China; CAS Key Laboratory of Pathogenic Microbiology and Immunology, Institute of Microbiology, Chinese Academy of Sciences, Beijing100101, China; Savaid Medical School, University of Chinese Academy of Sciences, Beijing100049, China; CAS Key Laboratory of Pathogenic Microbiology and Immunology, Institute of Microbiology, Chinese Academy of Sciences, Beijing100101, China; CAS Key Laboratory of Pathogenic Microbiology and Immunology, Institute of Microbiology, Chinese Academy of Sciences, Beijing100101, China; College of Veterinary Medicine, Shanxi Agricultural University, Jinzhong030801, China; Savaid Medical School, University of Chinese Academy of Sciences, Beijing100049, China; CAS Key Laboratory of Pathogenic Microbiology and Immunology, Institute of Microbiology, Chinese Academy of Sciences, Beijing100101, China; College of Veterinary Medicine, Shanxi Agricultural University, Jinzhong030801, China; Savaid Medical School, University of Chinese Academy of Sciences, Beijing100049, China

**Keywords:** marine animals, SARS-CoV-2, cross-species recognition, cryo-EM structure

## Abstract

Severe acute respiratory syndrome coronavirus 2 (SARS-CoV-2) has an extremely broad host range that includes hippopotami, which are phylogenetically closely related to whales. The cellular ACE2 receptor is one of the key determinants of the host range. Here, we found that ACE2s from several marine mammals and hippopotami could efficiently bind to the receptor-binding domain (RBD) of both SARS-CoV and SARS-CoV-2 and facilitate the transduction of SARS-CoV and SARS-CoV-2 pseudoviruses into ACE2-expressing cells. We further resolved the cryo-electron microscopy complex structures of the minke whale ACE2 and sea lion ACE2, respectively, bound to the RBDs, revealing that they have similar binding modes to human ACE2 when it comes to the SARS-CoV-2 RBD and SARS-CoV RBD. Our results indicate that marine mammals could potentially be new victims or virus carriers of SARS-CoV-2, which deserves further careful investigation and study. It will provide an early warning for the prospective monitoring of marine mammals.

## INTRODUCTION

Finding out the origin, together with the host range, of a virus causing emerging and re-emerging infectious diseases, is vital, and may provide guidance for disease control, and understanding of virus spillover in the future [1,2]. The current coronavirus disease 2019 (COVID-19) pandemic is caused by the infection of severe acute respiratory syndrome coronavirus 2 (SARS-CoV-2), which belongs to the betacoronavirus (BetaCoV) genus of the family *Coronaviridae*. This family consists of four genera: alphaCoV, betaCoV, gammaCoV and deltaCoV. Since its identification, natural infections of SARS-CoV-2 in various species have been reported in multiple animals, including cats, dogs, minks, tigers, African lions, ferrets, pumas, gorillas, snow leopards, white-tailed deers [3,4] and, most recently, hippopotami (https://www.bbc.com/news/world-europe-59516896).

Additionally, salmon carcasses were suspected to be carriers of SARS-CoV-2, and triggered a local outbreak of COVID-19 in Beijing, China in June 2020 [5]. It is reported that the surfaces of salmon carcasses were found to be SARS-CoV-2-positive during cold-chain transportation in Xinfadi Market [5]. However, no live SARS-CoV-2 was isolated and the role of salmon carcasses in the outbreak remains elusive. In another report, it was found that a live virus was isolated from cold-chain materials, including seafood [5,6]. These events a reminders that additional attention should be paid to marine animals, since the ocean covers 70% of the Earth's surface and harbors a huge number of lives, from the shoreline to the deepest sea floor. Marine mammals carry coronaviruses (CoVs). The first one was identified in a beluga whale in 2008 and was genetically related to gammaCoV [7]. In 2013, Yuan *et al.* identified another CoV in the bottlenose dolphin that resembles the beluga whale CoV and proposed that they be classified as a distinct species, *Cetacean coronavirus* [8]. Marine mammals are sentinels for oceans and human health. If SARS-CoV-2 spreads to marine mammals and circulates among marine species, it would pose an ‘underwater’ threat to pandemic control.

Before the outbreak of COVID-19, another CoV, severe acute respiratory syndrome coronavirus (SARS-CoV), caused an epidemic with a short duration two decades ago [9]. Since its outbreak, extensive research has been conducted into its origin and interspecies transmission [10,11]. SARS-CoV and SARS-CoV antibodies were detected in masked palm civets (*Paguma larvata*) and animal handlers in a market [12,13], suggesting civets might be intermediate hosts. However, subsequent wide-ranging investigations into farmed and wild civets indicated that they were clear of SARS-CoV, and SARS-CoV was more likely to have been transmitted to them from other natural hosts or even from humans [13,14]. Reports on novel CoVs closely related to SARS-CoV (SARSr-CoVs) in horseshoe bats indicate that bats might be the natural hosts [15,16]. Host adaptation of the spike (S) protein in SARS-CoV isolated from humans or civets has consistently been reported, with K479N and S478T being the key changes for adapting to the human receptor [10,17]. Similarly to SARS-CoV-2, SARS-CoV also has a broad potential host range. Strangely, no further case of infection has been reported after the SARS epidemic in 2004 [18]. Where SARS-CoV went remains a mystery.

CoV infection is initiated by the binding of the S protein on the viral particle to host surface cellular receptors. SARS-CoV-2 uses the receptor-binding domain (RBD) in the S protein to interact with angiotensin-converting enzyme 2 (ACE2), which is also the receptor of SARS-CoV [19]. The gain-of-function of a virus to bind to receptor orthologs in other species is a prerequisite for inter-species transmission. Reciprocally, screening receptor orthologs from different species for interaction with the S protein or RBD would narrow down the susceptible intermediate or natural hosts. By comparative analysis of ACE2 orthologs, especially the cross-species conservation of the key residues participating in the interaction between ACE2 and RBDs, several studies have predicted the potential host range of SARS-CoV-2 in multiple species by evaluating the binding propensity [4,20–24]. The results are highly consistent with the known natural infection host range. Among these predictions, many marine mammals (27/36) are predicted to have a high or very high risk of SARS-CoV-2 infection [23]. However, a real binding test and a pseudovirus infection assay concentrating on marine mammals are elusive, and deserve further analysis.

In this study, we tested the binding capacities of the SARS-CoV-2 RBD and SARS-CoV RBD to nine typical marine species, including both mammals and salmonids. We found that ACE2s from sperm whales, minke whales, dolphins, sea lions, fur seals and hippopotami could efficiently bind to the RBD of both SARS-CoV and SARS-CoV-2 and facilitate the transduction of SARS-CoV and SARS-CoV-2 pseudoviruses into ACE2-expressing cells. Then, we chose minke whale ACE2 and sea lion ACE2, which displayed high binding affinities to both RBDs, for further study and determined their cryo-electron microscopy (cryo-EM) structures in complex with either SARS-CoV-2 RBD or SARS-CoV RBD. Our work sheds light on the potential susceptibility of marine mammals to SARS-CoV-2 and highlights the need to strengthen surveillance of marine mammals to prevent potential spillovers and transmission.

## RESULTS

### Binding capacity of SARS-CoV-2 and SARS-CoV RBDs to marine animal ACE2 orthologs and pseudovirus transduction mediated by their interactions

**Figure 1.**
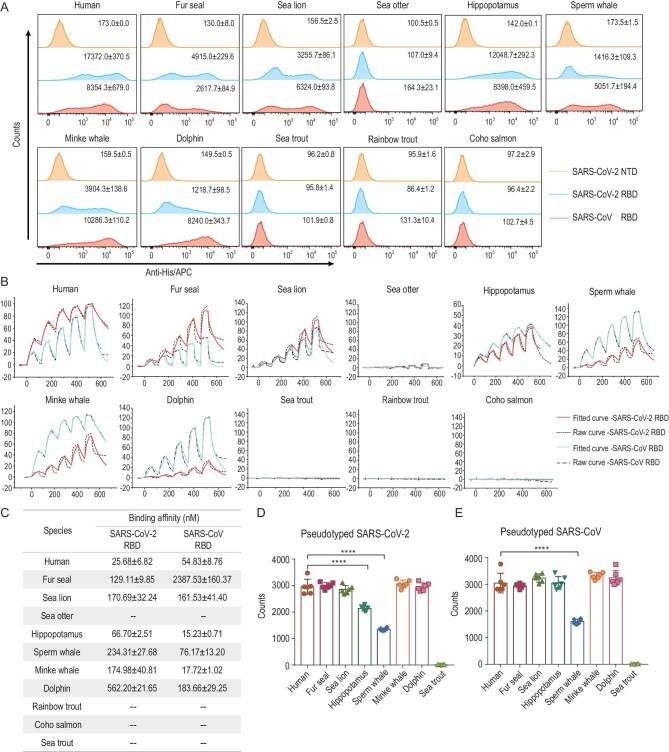
Binding between ACE2s and SARS-CoV-2 or SARS-CoV RBD, and the transduction of pseudotyped SARS-CoV-2 or pseudotyped SARS-CoV into BHK-21 cells expressing the relevant ACE2s. (A) His-tagged SARS-CoV-2 RBD, SARS-CoV RBD or SARS-CoV-2 N-terminal domain (NTD) proteins were incubated with BHK-21 cells expressing EGFP-tagged ACE2s, respectively. Anti-His/APC antibodies were used to detect the His-tagged protein binding to the cells. Cells stained with the SARS-CoV-2 RBD, the SARS-CoV RBD and the SARS-CoV-2 NTD proteins are shown in bright blue, pink and brown, respectively. The mean fluorescence values of APC are presented. The SARS-CoV-2 NTD was used as the negative control. (B) The mFc-tagged ACE2s in the supernatants were captured by anti-mIgG Fc antibodies immobilized on the CM5 chip, and their binding was sequentially tested with serially diluted SARS-CoV-2 RBD or SARS-CoV RBD. The raw and fitted curves are displayed in dotted and solid lines, respectively. (C) The binding affinities between ACE2s and SARS-CoV-2 RBD or SARS-CoV RBD are shown as the means ± SD of three independent experiments. (D and E) Transduction of the pseudotyped SARS-CoV-2 and SARS-CoV on BHK-21 cells expressing the respective mammal ACE2 or hACE2. Error bars represent the SD from six replicates. *P* values were analyzed using the student's t test (*** *P* < 0.001, **** *P* < 0.0001).

To evaluate the susceptibility of marine mammals to SARS-CoV and SARS-CoV-2, we chose six animals covering representative species of marine mammals: sperm whale, minke whale, dolphin, fur seal, sea lion and sea otter (Supplementary Fig. 1). We also included three salmonids, as salmon carcasses have been found to be SARS-CoV-2-positive during cold-chain transportation [5,6]. Additionally, animals that have been reported to have been naturally infected with SARS-CoV-2 (gorillas, cats, dogs, tigers, ferrets, minks, white tailed deer and hippopotami), and those with clear binding mechanisms to the SARS-CoV-2 RBD (Malayan pangolin and big-eared horseshoe bat), as well as masked palm civets and the identified bat reservoir species for SARS-CoV [25], were also included to investigate their evolutionary relationships. Based on ACE2 sequences, a phylogenetic tree was constructed (Supplementary Fig. 1). Dogs, cats, sea otters, fur seals and sea lions are all classified as members of the order *Carnivora*. Whales and hippopotami belong to the order *Artiodactyla*. We highlighted the SARS-CoV-2- and SARS-CoV-RBD-interacting residues on hACE2 [19], respectively, and compared them among the selected species (Supplementary Figs 1 and 2). Compared to hACE2, the number of amino acid changes in ACE2 orthologs from mammals ranges from 0 to 16, whereas those of salmonid ACE2 orthologs are 15 or 16, reflecting a large evolutionary distance. Among the binding site residues, S19, F28, D355, R357 and R393 are completely conserved. F28, as explained in our previous report [21], forms a hydrophobic interaction with Y83/F83 and stabilizes the two N-terminal helices of ACE2. When salmonids and masked palm civets were excluded, E37 and K353 were also strictly conserved. We conducted fluorescence-activated cell sorting (FACS) and surface plasmon resonance (SPR) assays to test the binding capacity of marine animal ACE2 orthologs to either SARS-CoV-2 RBD or SARS-CoV RBD. The FACS results demonstrate that both RBDs could bind to ACE2 orthologs from the selected marine animals, except for sea otter and salmonid ACE2s (Fig. 1A). The percentage of RBD-bound ACE2-expressing cells is shown in Supplementary Fig. 3. SPR assay results were consistent with the two RBDs displaying no binding to salmonid ACE2s. However, marine mammal and hippopotamus ACE2s, except for the sea otter ACE2, all had high binding affinities. Notably, MW-ACE2 showed even higher binding affinity with SARS-CoV RBD than hACE2 (Fig. 1B and C).

Next, to test the ability of ACE2 orthologs to support viral invasion, we transfected BHK-21 cells with plasmids containing enhanced green fluorescent protein (EGFP)-tagged ACE2s from marine mammals, hippopotami and sea trout, respectively. The EGFP-positive cells were sorted into 96-well plates and then incubated with pseudotyped SARS-CoV-2 and SARS-CoV, respectively. As a result, ACE2s from these marine mammals supported SARS-CoV-2 and SARS-CoV pseudotyped virus entry into ACE2-expressing BHK-21 cells, whereas sea trout ACE2 did not (Fig. 1D). Notably, hippopotamus ACE2 readily mediated the entry of both pseudoviruses.

### Overall architectures of both MW-ACE2 and SL-ACE2 bound to either the SARS-CoV-2 RBD or SARS-CoV RBD

**Figure 2.**
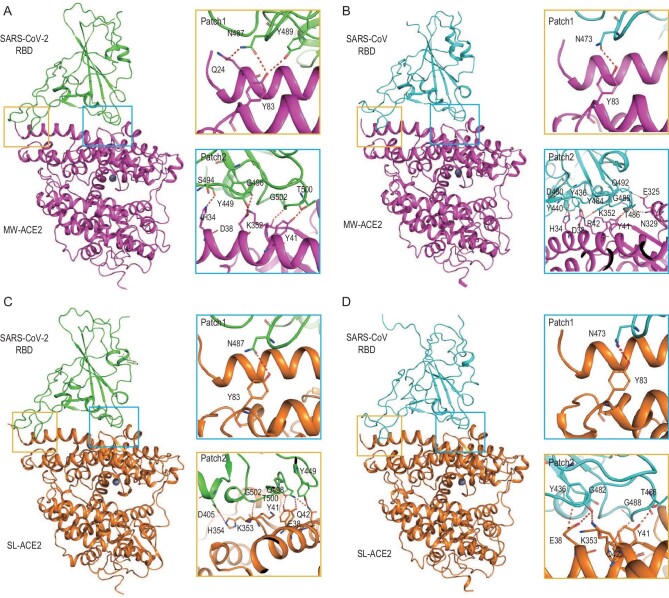
Overall architectures of the MW-ACE2/SARS-CoV-2 RBD, MW-ACE2/SARS-CoV RBD, SL-ACE2/SARS-CoV-2 RBD and SL-ACE2/SARS-CoV RBD complexes. Overall structure of the (A) MW-ACE2/SARS-CoV-2 RBD, (B) MW-ACE2/SARS-CoV RBD, (C) SL-ACE2/SARS-CoV-2 RBD and (D) SL-ACE2/SARS-CoV RBD complexes. Boxes indicate the interaction patches. The hydrogen bonds network of Patch 1 and Patch 2 are shown. A cartoon representation of the complex structure is shown, and residues participating in hydrogen bond formation are shown as sticks.

To reveal the molecular mechanisms behind the high-affinity binding of marine mammal ACE2s to SARS-CoV-2 RBD or SARS-CoV RBD, we chose MW-ACE2 and SL-ACE2, which had the highest binding affinities with both RBDs. Further, these species also represent two types of high-risk mammals: whales, which live social lives, thus enabling virus circulation within their population; and sea lions, which live near the coast and are at risk of contact with humans and coastal birds or bats. We prepared complexes of MW-ACE2/SARS-CoV-2 RBD, MW-ACE2/SARS-CoV RBD, SL-ACE2/SARS-CoV-2 RBD and SL-ACE2/SARS-CoV RBD, and solved their structures at resolutions of 2.87, 2.93, 3.03 and 2.89 Å, respectively (Supplementary Table 1). MW-ACE2/SARS-CoV-2 RBD and SL-ACE2/SARS-CoV-2 RBD display similar architectures to hACE2 in complex with SARS-CoV-2 RBD [19,26], with root mean squared deviations (RMSDs) of 0.995 Å and 0.594 Å, respectively (Fig. 2A and C). However, the MW-ACE2/SARS-CoV RBD and SL-ACE2/SARS-CoV RBD complexes showed more divergent architecture with hACE2 complexed with SARS-CoV RBD, with RMSD of 1.598 Å and 2.069 Å, respectively (Fig. 2B and D).

The external subdomains of SARS-CoV-2 RBD and SARS-CoV RBD, consisting of a loop between two short beta-sheets, are responsible for ACE2 recognition. The total numbers of interactions between the RBDs of SARS-CoV-2 and SARS-CoV, respectively, with MW-ACE2 and SL-ACE2, are 307, 285, 280 and 286, including 8, 10, 7 and 7 H-bonds, respectively (Supplementary Table 2 and 3). Notably, MW-ACE2 forms significantly more van der Waals (vdw) contacts and comparable H-bonds with SARS-CoV RBD than hACE2, which explains its stronger binding to the SARS-CoV RBD.

The interacting residues on the SARS-CoV-2 RBD and SARS-CoV RBD in the four complexes, similar to those in the hACE2/SARS-CoV-2 RBD complex [19], can be divided into two patches. In patch 1, Q24 and Y83 on MW-ACE2 form an H-bond network with N487 and Y489 on the SARS-CoV-2 RBD in the MW-ACE2/SARS-CoV-2 RBD complex (Fig. 2A), whereas in the other three complexes, only Y83 forms an H-bond with N473 or N487 (Fig. 2B–D). In patch 2, H34, D38, Y41 and K352 (corresponding to K353 in hACE2) on MW-ACE2 form an H-bond network with S494, Y449, T500, G496 and G502 on the SARS-CoV-2 RBD, respectively. When bound to the SARS-CoV RBD, R42 (corresponding to Q42 in hACE2), E325 (corresponding to G326 in hACE2) and N329 (corresponding to N330 in hACE2) on MW-ACE2 form additional H-bonds (Fig. 2A and B). In comparison, E38, Y41, Q42, K353 and H354 on SL-ACE2 form an H-bond network with Y449 and Q498, T500, Y449, G502 and D405 on the SARS-CoV-2 RBD, respectively, while H354 on SL-ACE2 is not involved in an H-bond network with the SARS-CoV RBD (Fig. 2C and D).

**Figure 3.**
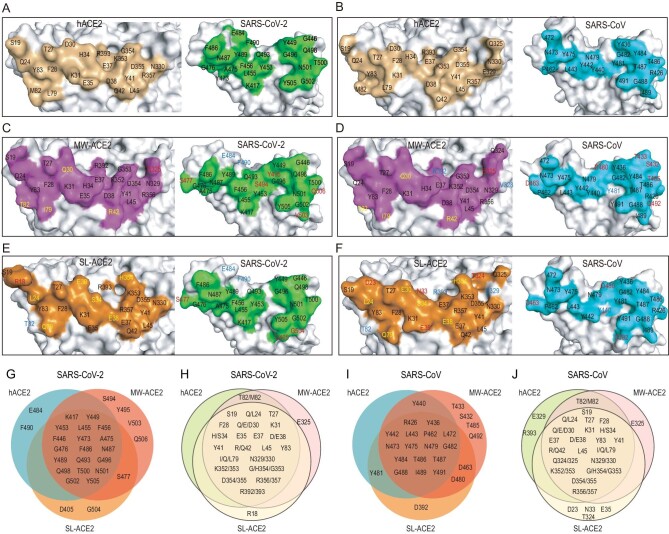
Interface comparison among RBDs of SARS-CoV-2 and SARS-CoV with ACE2 orthologs. Binding interface of (A) hACE2/SARS-CoV-2 RBD, (B) hACE2/SARS-CoV RBD, (C) MW-ACE2/SARS-CoV-2 RBD, (D) MW-ACE2/SARS-CoV RBD, (E) SL-ACE2/SARS-CoV-2 RBD and (F) SL-ACE2/SARS-CoV RBD. Venn diagrams of key residues on (G) SARS-CoV-2 RBD and (I) SARS-CoV RBD that are involved in the interaction with the three ACE2s. Key residues on hACE2, MW-ACE2 and SL-ACE2 participate in the interaction with (H) SARS-CoV-2 RBD and (J) SARS-CoV RBD. In panels C–F, residues in blue indicate that they are only involved in RBD binding by hACE2 but not in the referred ACE2 ortholog. Residues in red indicate that they are only involved in the RBD interaction of the referred ACE2 ortholog but not in hACE2. Residues in yellow indicate that a substitution was observed on the ACE2 interface compared with hACE2.

We then compared the interface residues among the six complexes: hACE2/SARS-CoV-2 RBD, hACE2/SARS-CoV RBD, MW-ACE2/SARS-CoV-2 RBD, MW-ACE2/SARS-CoV RBD, SL-ACE2/SARS-CoV-2 RBD and SL-ACE2/SARS-CoV RBD. When bound to the SARS-CoV-2 RBD, MW-ACE2 exhibits four substitutions compared to hACE2, namely D30Q, Q42R, L79I and M82T. E325 in MW-ACE2 is involved in RBD recognition, whereas the counterpart G326 on hACE2 is not (Fig. 3A, C and H). On the SARS-CoV-2 RBD, E484 and F490 exclusively interact with hACE2, while S477, S494, Y495, V503 and Q506 only interact with MW-ACE2 but not hACE2 (Fig. 3A, C and G). Regarding the SL-ACE2 interface, seven substitutions are found, namely Q24L, D30E, H34S, D38E, L79Q, M82T and G354H. M82 on hACE2 interacts with the SARS-CoV-2 RBD, whereas its sea lion counterpart (T82) is irrelevant (Fig. 3E and H). Notably, R18 on SL-ACE2 participates in the SARS-CoV-2 RBD interaction. E484 and F490 on the SARS-CoV-2 RBD interact with hACE2, and S477, D405 and G504 interact with SL-ACE2 but not with hACE2 (Fig. 3A, E and H).

When bound to the SARS-CoV RBD, MW-ACE2 shows five substitutions, including D30Q, Q42R, L79I, M82T and E329V (328 in MW-ACE2 numbering). V328 (329 in hACE2 numbering) and R392 (393 in hACE2 numbering) are no longer involved in RBD recognition (Fig. 3B, D and J). On the SARS-CoV RBD, S432, T433, D463, D480, T485 and Q492 interact with MW-ACE2 but not with hACE2, and Y481 exclusively interacts with hACE2 (Fig. 3B, D and I). Where SL-ACE2 is concerned, the same seven substitutions are also observed. D23, N33, E35 and T324 on SL-ACE2, but not on hACE2, interact with the SARS-CoV RBD (Fig. 3B, F and J). M82, E329 and R393 on hACE2 are involved in the interaction while their counterparts on SL-ACE2 are not (Fig. 3J). When comparing the interface residues of SL-ACE2/SARS-CoV RBD and hACE2/SARS-CoV RBD complexes, Y440 on the SARS-CoV RBD exclusively binds to hACE2 while D463 and D480 bind to SL-ACE2 (Fig. 3B, F and I).

### Distinctive binding sites on MW-/SL-/hACE2 in complex with the SARS-CoV-2 RBD and SARS-CoV RBD

**Figure 4.**
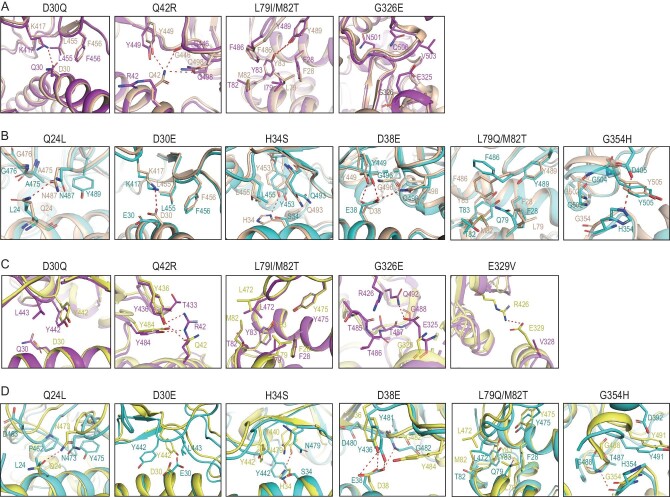
Structural details of MW-ACE2/SARS-CoV-2 RBD, MW-ACE2/SARS-CoV RBD, SL-ACE2/SARS-CoV-2 RBD and SL-ACE2/SARS-CoV RBD. (A and B) Structural alignment of hACE2/SARS-CoV-2 RBD (wheat) with MW-ACE2/SARS-CoV-2 RBD (purple, A), or with SL-ACE2/SARS-CoV-2 RBD (cyan, B). Substitutions of hACE2 with MW-ACE2 or SL-ACE2 are labeled above the figure. Residues involved in the interaction are presented as sticks, and polar interactions are presented by red dashes. (C and D) Structural alignment of hACE2/SARS-CoV RBD (yellow) with MW-ACE2/SARS-CoV-2 RBD (purple, C) or with SL-ACE2/SARS-CoV-2 RBD (cyan, D). Substitutions of hACE2 with MW-ACE2 or SL-ACE2 are labeled above the figure. Residues involved in the interaction are presented as sticks and polar interactions are presented by red dashes.

To analyze the effect of MW-ACE2 and SL-ACE2 substitutions on the RBDs’ interactions, we aligned the α1 and α2 helices of the MW-ACE2/SARS-CoV-2 RBD and SL-ACE2/SARS-CoV-2 RBD complexes to helices of the hACE2/SARS-CoV-2 RBD complex, respectively. Five substitutions were observed on the MW-ACE2 binding interface to SARS-CoV-2 RBD, compared to hACE2, namely D30Q, Q42R, L79I, M82T and G326E (Fig. 4A). Structural analysis indicated that D30Q loses the salt bridge between K417 and D30, which may lead to decreased binding affinity. The side chain of R42 on MW-ACE2, though much longer than Q42 on hACE2, is bent away from its interacting residues on the SARS-CoV-2 RBD and fails to form any polar interactions, while Q42 on hACE2 forms three H-bonds with the RBD (Supplementary Fig. 5). On hACE2, both L79 and M82 participate in the hydrophobic patch consisting of Y489 and F486 in the SARS-CoV-2 RBD and F28, L79, M82 and Y83 on hACE2 [19]. Substitution of M82 with T82 (hydrophilic) might undermine the hydrophobic patch, while that of L79 with I79 should not have a significant impact. Substitution of G326 on hACE2 with its MW-ACE2 counterpart E325 brings with it a longer side chain, which forms additional interactions with the RBD and expands the binding interface (Fig. 4A).

The SARS-CoV-2 RBD binding interface of SL-ACE2 carries seven substitutions (Fig. 4B). As a polar amino acid, Q24 of hACE2 forms a new H-bond with N487, which may strengthen the RBD binding. The Q24L substitution decreases the binding of SL-ACE2 with the SARS-CoV-2 RBD, and both D30E and D38E confer longer side chains. However, the side chain of E30 is bent and does not form a salt bridge with K417, while E38 forms an additional H-bond with Q498. H34 of SL-ACE2 forms an H-bond with Y453 and may facilitate RBD binding. The M82T substitution plays a similar role in the hydrophobic patch to that of M82T in the MW-ACE2/SARS-CoV-2 RBD complex, and L79Q, with its longer chain and polarity, might undermine the hydrophobic patch. Thus, the conformation of F486 on SL-ACE2 is shifted compared to the counterpart residue on hACE2. Transition from G354 to H354 confers an additional salt bridge with D405, strengthening the interaction.

When binding to the SARS-CoV RBD, MW-ACE2 involves another substitution, E329V, which negates a salt bridge between E329 and R426 (Fig. 4C). Notably, R42 of MW-ACE2 forms two H-bonds with Y436 and Y484 of the SARS-CoV RBD, and E325 of MW-ACE2 forms an H-bond with Q492. However, its counterpart G326 does not participate in RBD binding. L79I and M82T function similarly in the hydrophobic patch to those in the MW-ACE2/SARS-CoV-2 RBD complex (Fig. 4C). The impact of substitutions in SL-ACE2 on the SARS-CoV RBD interaction is basically similar to that of the SARS-CoV-2 RBD (Fig. 4D). Notably, the main chain of H354 of SL-ACE2 forms an H-bond with G488 on the SARS-CoV RBD while G354 on hACE2 does not, which might result from alteration of loop positioning.

## DISCUSSION

CoVs such as SARS-CoV, SARS-CoV-2, RaTG13, GD/1/2019 and GX/P2V/2017 broadly recognize different ACE2 orthologs [27,28]. SARS-CoV-2 has been reported to infect 14 species in nature so far [4]. Recently, two hippopotami have tested positive for SARS-CoV-2 at Antwerp Zoo in Belgium, and are the first reported cases in this species (https://www.bbc.com/news/world-europe-59516896). However, the source of infection is unknown, though human transmission is suspected. Notably, genome evidence and phylogenetic analyses suggest that hippopotami are close living relatives of whales. The natural infection of hippopotami suggests that whales and other marine mammals may be in danger of SARS-CoV-2 infection, especially the ones in zoos, which have much more contact with human beings than those in nature. Therefore, this led to our hypothesis for this study.

Here, we identified that the ACE2s of whales, dolphins, fur seals, sea lions and hippopotami could efficiently bind to both SARS-CoV-2 and SARS-CoV RBD proteins with different affinities. The MW-ACE2 bound tighter to the SARS-CoV RBD than to the hACE2 receptor. In contrast, the ACE2s from sea trout, rainbow trout and Coho salmon, which belong to the order *Salmoniformes*, did not bind to either viral RBD. Moreover, the pseudoviruses incorporating the SARS-CoV-2 or SARS-CoV S protein could also invade marine mammal, but not salmonid, ACE2-expressing BHK-21 cells. This indicates that whales, dolphins, fur seals and sea lions are all at high risk of SARS-CoV-2 or SARS-CoV virus infection.

Previous data by other groups, as well as ours, reported the binding between SARS-CoV-2 RBD and ACE2 in a broad range of animals, including dogs and cats, in line with the epidemiology observation that both dogs and cats are susceptible to SARS-CoV-2 infection [21,29,30]. However, there are differences between house pets (dogs and cats) and marine mammals. Cats and dogs engage with human life, while marine mammals live in the ocean and only occasionally come into contact with human beings. These occasional contacts include visitors to coastal pinnipeds meeting with sea lions, for instance. In some countries, whale hunting is still permitted, thus increasing the opportunity for these animals to come into contact with infected people.

Aside from direct contact with infected individuals, the viral shedding of SARS-CoV-2 in the feces and urine of infected individuals were also reported. This infectious-particle-containing raw sewage usually ends up in rivers, lakes or even the sea. Supportively, SARS-CoV-2 can be detected in untreated wastewater and in rivers [31–33], making waste a potential source of person-to-person transmission and environment-based spreading of COVID-19 [34,35], and increasing the opportunity for marine mammals to be infected by the virus expelled into the sea. Notably, the discharge of SARS-CoV-2-containing feces from shipping vessels with SARS-CoV-2-infected individuals, into the sea, may increase the infection risk for these mammals. Thus, SARS-CoV-2-contaminated waste water is probably an emerging concern with regard to the exposure of marine mammals to the virus. It is worth noting that marine mammals in zoos are an exception, as they have much more contact with human beings. Although no marine mammal has been reported as being infected to date, our results indicate the necessity of taking measures to inactivate the virus in waste water, if there is any, and strengthen virus surveillance on these marine mammals, especially the ones in zoos.

When water is considered a potential vehicle for spreading the virus, one obvious question is the stability of SARS-CoV-2 in fresh and salt water. Casanova *et al.* found that the time required for a 99% reduction in reagent-grade water is 22 days for the transmissible gastroenteritis virus (TGEV) and 17 days for mouse hepatitis virus (MHV) at room temperature, both of them CoV members. In pasteurized settled sewage, the time required for a 99% reduction is 9 days for TGEV and 7 days for MHV [36]. One modeling study suggests that SARS-CoV-2 viral particles may be stable for more than 25 days [37] but another study indicates that infectious SARS-CoV-2 is stable in river water for 2.3 days and 3.8 days at 20°C and 4°C, respectively. In seawater, the decay time is 1.1 days and 2.2 days at 20°C and 4°C, respectively [38]. There is no doubt that real environmental conditions are much more complicated, but the fact that other CoVs can be found in marine mammals indicates that CoVs are stable enough for viral transmission in or across species in the sea [7,8].

Previously, cross-species transmission of viruses has been suggested, both between marine mammals and between humans and marine mammals [39–41]. Marine mammals are widely reported to host the influenza virus [42]. The influenza A (H1N1) virus has been detected in sea otters and seals. Of sea otters captured off the Washington coast, 70% tested positive for H1N1 IgG antibodies in one study, though the origin and transmission route remain unknown [43]. However, seals and humans are the only known hosts of influenza B viruses [44]. In another study, an H7N7 infected seal sneezed directly into the face and right eye of one researcher, which caused severe conjunctivitis in the exposed person. However, no virus was isolated and no specific antibodies were detected [41]. A subsequent animal experiment revealed that intratracheal administration of seal influenza virus in non-human primates could induce symptoms almost similar to those of a human influenza A virus infection [45].

Although there are some substitutions in WM-ACE2 and SL-ACE2 compared to hACE2, SARS-CoV and SARS-CoV-2 can also bind to these two ACE2s with binding affinities comparable to hACE2. The cryo-EM complex structures of MW-ACE2/SARS-CoV-2 RBD, MW-ACE2/SARS-CoV RBD, SL-ACE2/SARS-CoV-2 RBD and SL-ACE2/SARS-CoV RBD revealed molecular information about the receptor binding of SARS-CoV-2 and SARS-CoV to two representative marine mammals. Q42 on hACE2 plays a critical role in the formation of the H-bond interaction network with the SARS-CoV-2 RBD. Herein, the Q42R substitution in MW-ACE2 has different consequences in the MW-ACE2/SARS-CoV-2 RBD and MW-ACE2/SARS-CoV RBD complexes. In the MW-ACE2/SARS-CoV-2 RBD complex, R42 does not form an H-bond with the SARS-CoV-2 RBD, on the contrary, Q42 in hACE2 forms three H-bonds with the SARS-CoV-2 RBD. In the MW-ACE2/SARS-CoV RBD complex, R42 of MW-ACE2 forms two H-bonds with Y436 and Y484 of SARS-CoV-RBD, respectively, but Q42 on hACE2 only forms one H-bond. In the hACE2/SARS-CoV-2 RBD complex, F486 of the RBD forms strong hydrophobic interactions with L79, M82 and Y83 of hACE2 [19]. In the MW-ACE2/SARS-CoV-2 RBD complex, the L79I and M82T substitutions do not change the conformation of F486 on the SARS-CoV-2 RBD. However, in the SL-ACE2/SARS-CoV-2 RBD complex, L79Q and M82T change the hydrophobic region to hydrophilic and push F486 away from the binding surface (Fig. 4B).

Additionally, successive SARS-CoV-2 variants, including the recently emerging Omicron variant, have rapidly burst onto the scene. The highly transmissible Omicron variant quickly became dominant. Our previous study has revealed the binding details of the Omicron (BA.1) RBD with hACE2 and found that the Omicron (BA.1) RBD binds to hACE2 at a similar affinity to that of prototype RBD [46]. In this study, we have evaluated the binding affinities between the Omicron BA.1 RBD and ACE2s of minke whales and sea lions, and the results are shown in Supplementary Fig. 6. The *K*_D_ value for BA.1 RBD binding to minke whale ACE2 is 253.67 ± 51.29 nM, which is lower than the prototype RBD (*K*_D_ = 155.50 ± 70.00 nM). In addition, BA.1 RBD lost the binding to the sea lion ACE2, suggesting a lower potential risk of inter-species transmission of SARS-CoV-2 Omicron than the prototype. The binding strength between other Omicron subvariants and marine mammal ACE2s needs further study. Regarding the latest subvariants of Omicron, BA.4 and BA.5, more studies are needed to evaluate their transmission and pathogenicity. Residues at sites 493, 498 and 501 are known to be the determinants of the host range [4]. Although an alignment of RBDs of Variants of concern (VOCs) can provide some information for predicting the binding of ACE2 to RBD (Supplementary Fig. 7), further studies are necessary to evaluate how easily viral infection in marine mammals can occur, as rapid viral mutations may break through the barriers between different species during the co-evolution of the virus and host.

Indeed, there are some limitations to this work. First, entry into cells through receptors is the first step of virus infection, which is a clue when studying the susceptibility of animals and inter-species transmission of the virus. Besides the receptor, many other viral and host factors also impact the host tropism of CoVs. For instance, transmembrane serine protease 2 (TMPRSS2) cleaves the S2 fusion machinery at the S2′ site to expose the fusion peptide and promote SARS-CoV-2 infection [47,48]. If the TMPRSS2 orthologs of these marine mammals have similar functions to those in humans, this would be another restrictive factor for inter-species transmission. Also, in this study, we applied pseudovirus-based transduction, a relative qualitative method, to evaluate the ability of specific ACE2s mediating the viral entry. The values are probably influenced by multiple factors, including the expression level of the ACE2 on the target cells. Thus, a likely contradiction was observed between the entry of the pseudovirus and the binding affinities, which was determined by SPR, a strictly quantitative evaluation method. It would be more accurate to evaluate infectivity of authentic virus in marine mammals and their cells. Even so, our study suggests the potential risk of infection in marine mammals for both SARS-CoV and SARS-CoV-2, and provides an early warning for the potential spillover and inter-species transmission of the viruses to marine mammals.

## MATERIALS AND METHODS

### SPR analysis

We tested the binding affinities between the mFc-tagged ACE2s and SARS-CoV-2 RBD or SARS-CoV RBD proteins by SPR using a BIAcore 8K (GE Healthcare) at 25°C in single-cycle mode. SARS-CoV-2 NTD protein was used as a negative control. The HBS-EP buffer (20 mM HEPES, pH 7.4, 150 mM NaCl and 0.005% (v/v) Tween 20) was used as the running buffer, and SARS-CoV-2 RBD, SARS-CoV RBD and SARS-CoV-2 NTD proteins were exchanged into this buffer by gel filtration before use. First, the anti-mFc antibodies were immobilized on the CM5 biosensor chip (GE Healthcare) using amine-coupling chemistry (GE Healthcare). Then, the supernatants containing mFc-tagged ACE2s were injected and captured respectively at ∼100–700 response units. SARS-CoV-2 RBD, SARS-CoV RBD or SARS-CoV-2 NTD protein was serially diluted and flowed through the chip surface and the binding response was measured. The anti-mFc antibody was regenerated with 10 mM Glycine-HCl (pH 1.7). The equilibrium dissociation constants (K_D_) of each interaction pair were calculated using BIAcore 8K Evaluation Software (GE Healthcare) by fitting to a 1 : 1 Langmuir binding model. Supernatant containing hACE2-mFc protein was used as a positive control.

### Cryo-EM sample preparation, data collection and image processing

For cryo-EM, the ACE2/RBD complex sample was vitrified using a Vitrobot Mark IV (Thermo Fisher Scientific) plunge freezing device. An aliquot sample was applied to a glow-discharged GO Quantifoil grid. The grid was then blotted for 0.5 sec with blot force set to 3 at a temperature of 4°C and a humidity level of >98%, and plunge frozen into liquid ethane.

For the four complexes, cryogenic specimens were loaded onto a 300 kV FEI Titan Krios transmission electron microscope for data collection. Micrographs were collected using EPU at 105 000×magnification (physical pixel size 0.67 Å) over a defocus range of −1.0 μM to −2.0 μM with a total accumulated dose of 60 e^−^/Å^2^.

The detailed data-processing workflow is summarized in Supplementary Figs 8–11. All of the raw dose-fractionated image stacks were 2×binned, aligned, dose-weighted and summed using MotionCor2. The contrast transfer function (CTF) estimation, particle picking and extraction, 2D classification, *ab initio* model generation, and 3D refinements were performed in cryoSPARC v.3.3.1.

### Model fitting and refinement

To model the four complexes, the atomic model of hACE2, bound to the SARS-CoV-2 RBD (Protein Data Bank (PDB) 6LZG), was fitted into the electron density map using Chimera. The initial structure model was refined against the cryo-EM density map in real space using Phenix, with secondary structure restraints. The model was manually corrected for local fit in COOT, and the sequence register was updated based on alignment. The stereochemical quality of the final model was assessed by MolProbity. The statistics for image processing and model refinement are summarized in Supplementary Table 1.

## DATA AVAILABILITY

The atomic coordinates for the crystal structures of the MW-ACE2/SARS-CoV-2 RBD (PDB code: 7WSE), MW-ACE2/SARS-CoV RBD (PDB code: 7WSF), SL-ACE2/SARS-CoV-2 RBD (PDB code: 7WSH) and SL-ACE2/SARS-CoV RBD (PDB code: 7WSG) complexes have been deposited in the PBD (https://www.rcsb.org/).

## Supplementary Material

nwac122_Supplemental_File
