# Supplementary material for: Cross-species recognition and molecular basis of SARS-CoV-2 and SARS-CoV binding to ACE2s of marine animals
Source: Natl Sci Rev. 2022 Jun 23;9(9):nwac122. doi: 10.1093/nsr/nwac122 (PMC9517163; doi:10.1093/nsr/nwac122)
Supplement: nwac122_Supplemental_File [file nwac122_supplemental_file.docx]

**SUPPLEMENTARY DATA**

**SUPPLEMENTARY MATERIALS AND METHODS**

**Gene cloning**

The SARS-CoV-2 RBD (residues 319–541, GISAID: EPI_ISL_402119), SARS-CoV-2 NTD (residues 20–286, GISAID: EPI_ISL_402119) and SARS-CoV RBD (residues 306–527, GenBank: NC_004718) used in this study were cloned into the pCAGGS vector and expressed using a mammalian cell expression system. The whole coding sequences of the 11 ACE2 orthologs (accession numbers are shown in Supplementary Table 4) were synthesized and cloned into the pEGFP-N1 vector, respectively, for FACS and pseudovirus infection assays. For SPR, the ectodomains of the 11 ACE2s fused with the Fc domain of mouse IgG (mFc) were individually cloned into the pCAGGS vector. The coding sequence of the WM- and SL-ACE2 extracellular domains were cloned into the pET21a vector for protein expression and purification.

**Protein expression and purification**

Protein expression and purification were performed following our previously reported methods [1], with some modifications. Briefly, for HEK293F cell expression, the pCAGGS plasmid containing the coding sequence of the SARS‐CoV‐2 RBD, SARS-CoV RBD or SARS-CoV-2 NTD was transiently transfected. After 72 h, the supernatant was collected, and soluble proteins were purified by using a HisTrap excel column (GE Healthcare), followed by gel filtration chromatography with a Superdex™ 200 Increase 10/300 GL column (GE Healthcare). For the mFc‐fusion protein expression in 293F cells, the culture supernatants containing the indicated proteins were collected and concentrated ~48 h after transfection. The pET21a-MW-ACE2 and pET21a-SL-ACE2 plasmids were transformed into *Escherichia coli* strain BL21 (DE3) for protein expression. MW-ACE2 and SL-ACE2 were over-expressed in E. coli as inclusion bodies and refolded. Briefly, the dissolved ACE2 inclusion bodies were diluted dropwise in a refolding buffer (100 mM Tris-HCl, pH 8.0, 2 mM EDTA, 400 mM L-arginine, 0.5 mM oxidized glutathione and 5 mM reduced glutathione) at 4 °C overnight. Then the refolded ACE2 proteins were concentrated using an Amicon 8400 concentrator with a 10- kDa cutoff membrane and exchanged into 20 mM Tris-HCl (pH 8.0) and 150 mM NaCl buffer and subsequently purified by gel-filtration chromatography with a HiLoad 16/600 SuperdexTM 200 pg column (GE Healthcare) using an ÄKTA System. To obtain the complexes of SARS-CoV RBD/MW-ACE2, SARS-CoV-2 RBD/MW-ACE2, SARS-CoV RBD/SL-ACE2 and SARS-CoV-2 RBD/SL-ACE2, purified ACE2s and the RBDs of the two CoVs (SARS-CoV and SARS-CoV-2) were mixed at a molar ration of 1:1.5. The two mixtures were incubated on ice for 2 h and further purified over Superdex^TM^ 200 Increase 10/300 GL column (GE Healthcare) with a buffer consisting of 20 mM Tris‐HCl (pH 8.0) and 150 mM NaCl. The four RBD-ACE2 complex peaks were collected and concentrated to 2 mg ml^−1^ for cryo‐electron microscopy (cryo‐EM) sample preparation, respectively.

**FACS analysis**

FACS analysis methods described in our previous study [2]. Briefly, BHK-21 cells (ATCC, ATCC CCL‐10) in 6-well-plates were transfected with pEGFP-N1-ACE2s plasmids, respectively. Then, 2 × 10^5^ cells were harvested in PBS after 24 h and incubated with the test proteins (SARS‐CoV‐2 RBD, SARS-CoV RBD and SARS‐CoV‐2 NTD) at 37°C for 30 min, which was followed by washing twice in PBS and staining for 30 min at 37°C with anti‐His/APC antibodies (1:500, Miltenyi Biotec, AB_2751870). FACS data were acquired on a BD FACSCanto and analyzed using FlowJo V10 software.

**Pseudovirus transduction**

The SARS‐CoV‐2 and SARS‐CoV pseudoviruses were constructed with an mCherry-encoding replication‐deficient vesicular stomatitis virus (VSV) vector backbone (VSV‐ΔG‐mCherry) and the coding sequence of the corresponding spike proteins, as previously described [3]. Briefly, HEK293T cells were transfected with spike protein expression plasmids. The VSV‐ΔG‐mcherry pseudovirus was added 24 h post‐transfection. The inoculum was removed after incubation for 2 h at 37°C. The culture medium was then changed into DMEM supplemented with 10% FBS and 10 μg ml^−1^ of anti‐VSV‐G antibody (I1‐Hybridoma ATCC CRL2700™) after washing the cells with PBS. The pseudoviruses were harvested 30 h post‐inoculation, passed through a 0.45‐μm filter before alquotting, and stored at -80°C. BHK-21 cells were transfected with each of the pEGFP-N1-ACE2s plasmids, and 24 h later, EGFP-positive cells were sorted and reseeded in 96-well plates at 2 × 10^4^ cells/well and cultivated for another 24 h. The BHK-21 cells were washed with PBS before the addition of the supernatant containing pseudovirus particles. Fifteen hours post-transfection, the imaging and analysis of the fluorescent cells was determined using a CQ1 confocal image cytometer (Yokogawa, Japan). Each group contained six replicates. BHK-21 cells transfected with pEGFP-N1-hACE2 were used as a positive control.

**Cryo-EM sample preparation**

For cryo-EM, the MW-ACE2/SARS-CoV RBD complex sample was vitrified using a Vitrobot Mark IV (Thermo Fisher Scientific) plunge freezing device. An aliquot of 4.0 μL (0.2 mg ml^−1^) sample was applied to a glow-discharged GO Quantifoil grid. The grid was then blotted for 0.5 sec with blot force set to 3 at a temperature of 4°C and a humidity level of >98% and plunge frozen into liquid ethane.

For the MW-ACE2/SARS-CoV-2 RBD complex, the SL-ACE2/SARS-CoV RBD complex, and SL-ACE2/SARS-CoV-2 RBD complex, cryogenic specimens were prepared following similar protocol. We use an Au Quantifoil grid to prepare the MW-ACE2/SARS-CoV-2 RBD complex cryogenic sample. The 4.0 μL complex sample (1.0 mg ml^−1^) was then applied on the grid, blotted with filter paper for 3.0 s with blot force set to 3 and plunge-frozen in liquid ethane. For the SL-ACE2/SARS-CoV RBD complex, sample (4.0 μL, 1.05 mg ml^−1^) was applied on an glow-discharged Au Quantifoil grid with blot time set to 2.0 sec and blot force set to 3. For the SL-ACE2/SARS-CoV-2 RBD complex, a 4.0 μL volume of 1.1 mg ml^−1^ sample was loaded onto a freshly glow discharged Au Quantifoil grid before plunge freezing using a blot force of 3 and 3.0 sec blot time at 100% humidity and 4 °C.

**Data collection and image processing**

For the four complexes, cryogenic specimens were loaded onto a 300 kV FEI Titan Krios transmission electron microscope for data collection. Micrographs were collected using EPU at 105,000× magnification (physical pixel size 0.67 Å) over a defocus range of -1.0 μM to -2.0 μM with a total accumulated dose of 60 e^-^/Å^2^.

The detailed data processing workflow is summarized in Supplementary Figures 8, 9, 10, and 11. All of the raw dose-fractionated image stacks were 2× binned, aligned, dose-weighted and summed using MotionCor2. The contrast transfer function (CTF) estimation, particle picking and extraction, 2D classification, *ab initio* model generation, and 3D refinements were performed in cryoSPARC v.3.3.1.

For the MW-ACE2/SARS-CoV RBD complex, a total of ~3,346 micrographs were collected for this dataset, from which initial particles were picked and subjected to 2D classification and 3D classification. After 2D classification, a subset of 476,095 particles were picked for 3D classification. The initial 3D model was also obtained from the 2D classes generated from this extended particle set. Among the six 3D classes, a dominant class containing ~31% of total particles was identified, which displayed clear features of secondary structural elements. These particles were subjected to 3D refinement, which yielded a final density map at 2.87 Å resolution estimated by the gold-standard Fourier shell correlation cut-off value of 0.143.

The MW-ACE2/SARS-CoV-2 RBD complex dataset was processed similarly. Initial particles were automatically picked from ~4,376 micrographs. After extensive 2D classification, approximately 817,599 good particles were selected to generate the initial model and 3D classification, resulting in three 3D classes. A single dominant class was identified, accounting for 54% of the input particles, which was used to calculate the final density map at 2.93 Å resolution.

For the SL-ACE2/SARS-CoV RBD complex, a total of ~4,417 micrographs were collected for this dataset, from which ~393,885 initial particles were picked. After multiple rounds of 2D classification and 3D classification, a clean subset of 117,386 good particles was isolated, which led to a reconstruction at 3.03 Å resolution.

For the SL-ACE2/SARS-CoV-2 RBD complex, a total of 4,006 micrographs were collected for this dataset, from which initial particles were picked and subjected to 2D classification and 3D classification. One round of reference-free 2D classification was performed to remove heterogeneous particles. A clean dataset with 539,017 particles from good 2D classes was selected and subjected to 3D classification. After the 3D classification, the predominant class containing a subset of the 183,322 best particles shows the best structural features and the highest accuracy of particle alignment, which was used for the 3D refinement and resulting in a final density map at a resolution of 2.89 Å.

**SUPPLEMENTARY FIGURES AND TABLES**

**
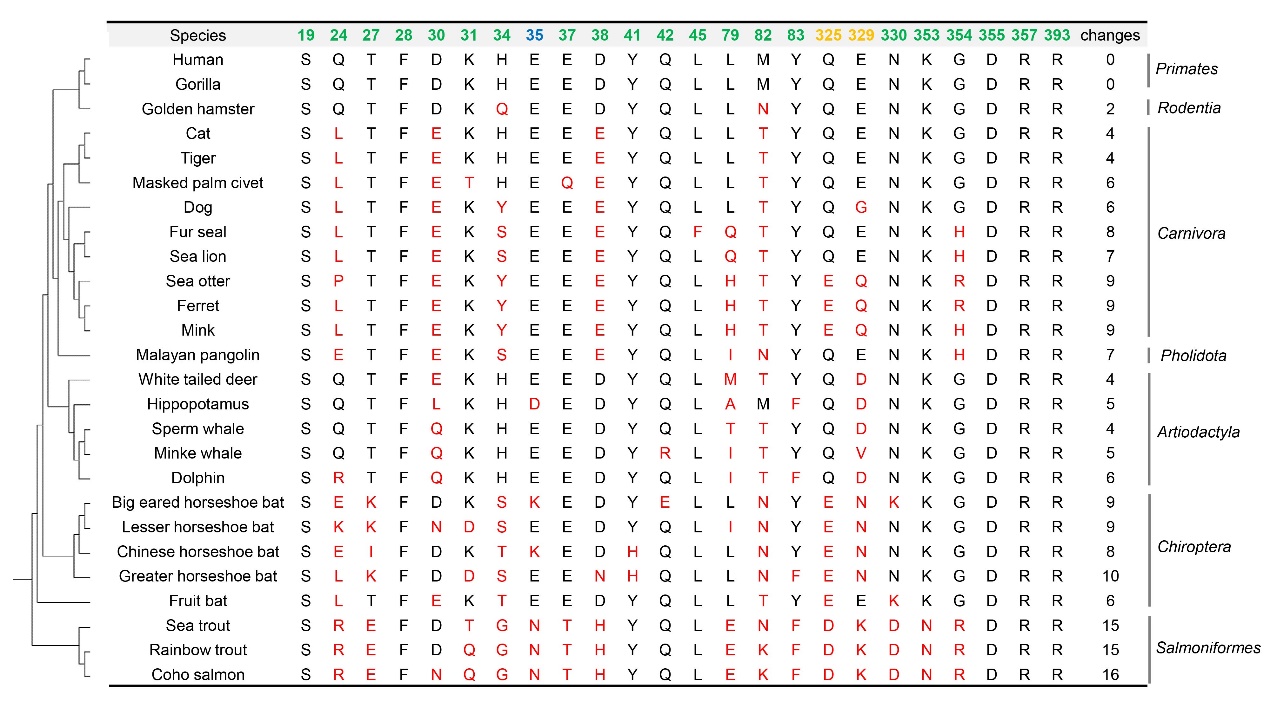
**

**Supplementary Figure 1.** Phylogenetic analysis of 26 animals based on ACE2 and characteristics of the SARS-CoV-2/SARS-CoV RBD-binding residues of ACE2s. The 26 species (including human) belonging to seven orders are shown in the right column. Residues of hACE2 that are crucial for the interaction with the SARS-CoV-2 RBD and SARS-CoV RBD are listed. Green numbers indicate contacts with both SARS-CoV RBD and SARS-CoV-2 RBD, and blue numbers indicate contacts with SARS-CoV-2 RBD only, orange numbers indicate contacts with SARS-CoV RBD only. Red letters indicate the amino acid changes in the ACE2 of the animal species compared with hACE2.


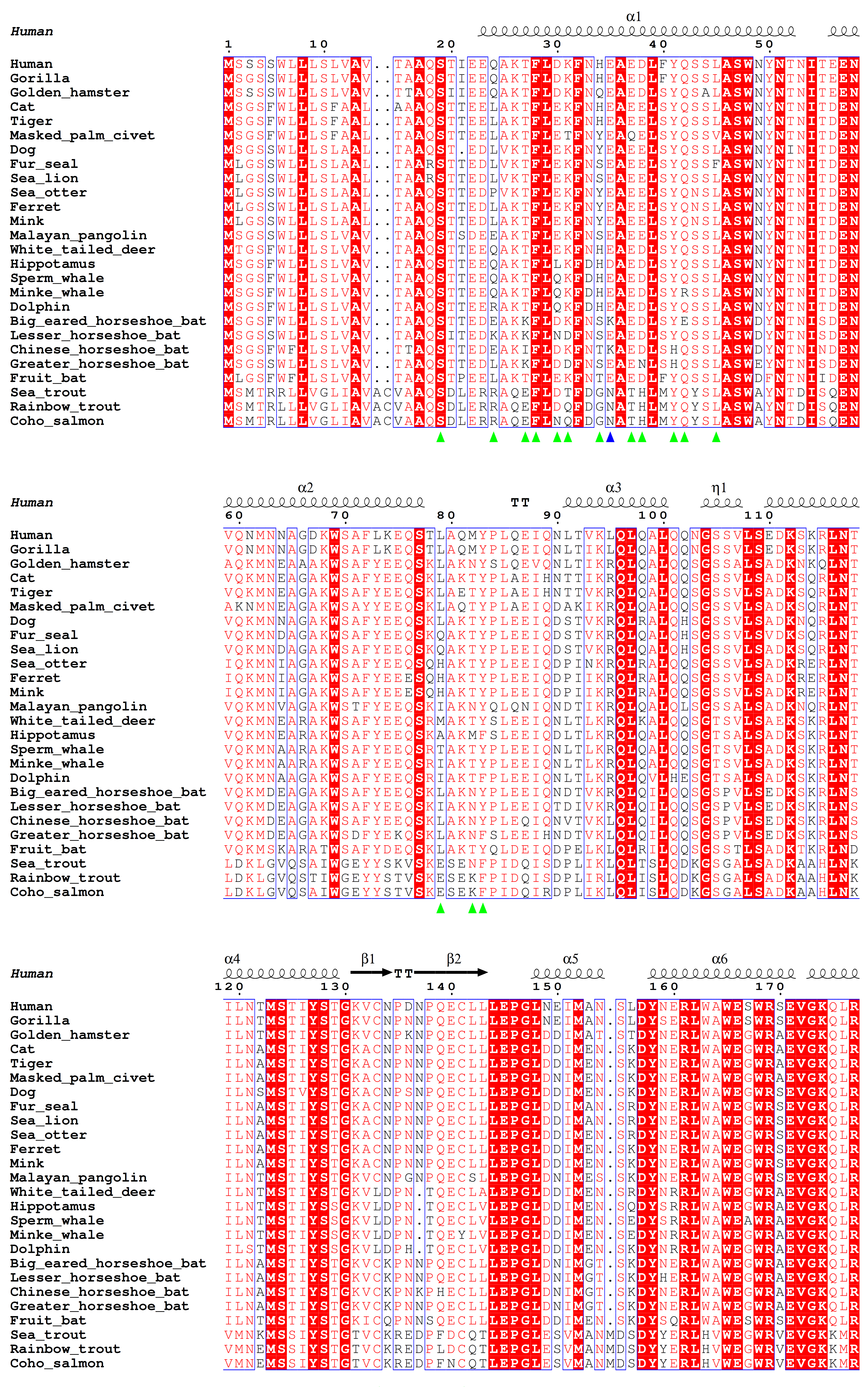


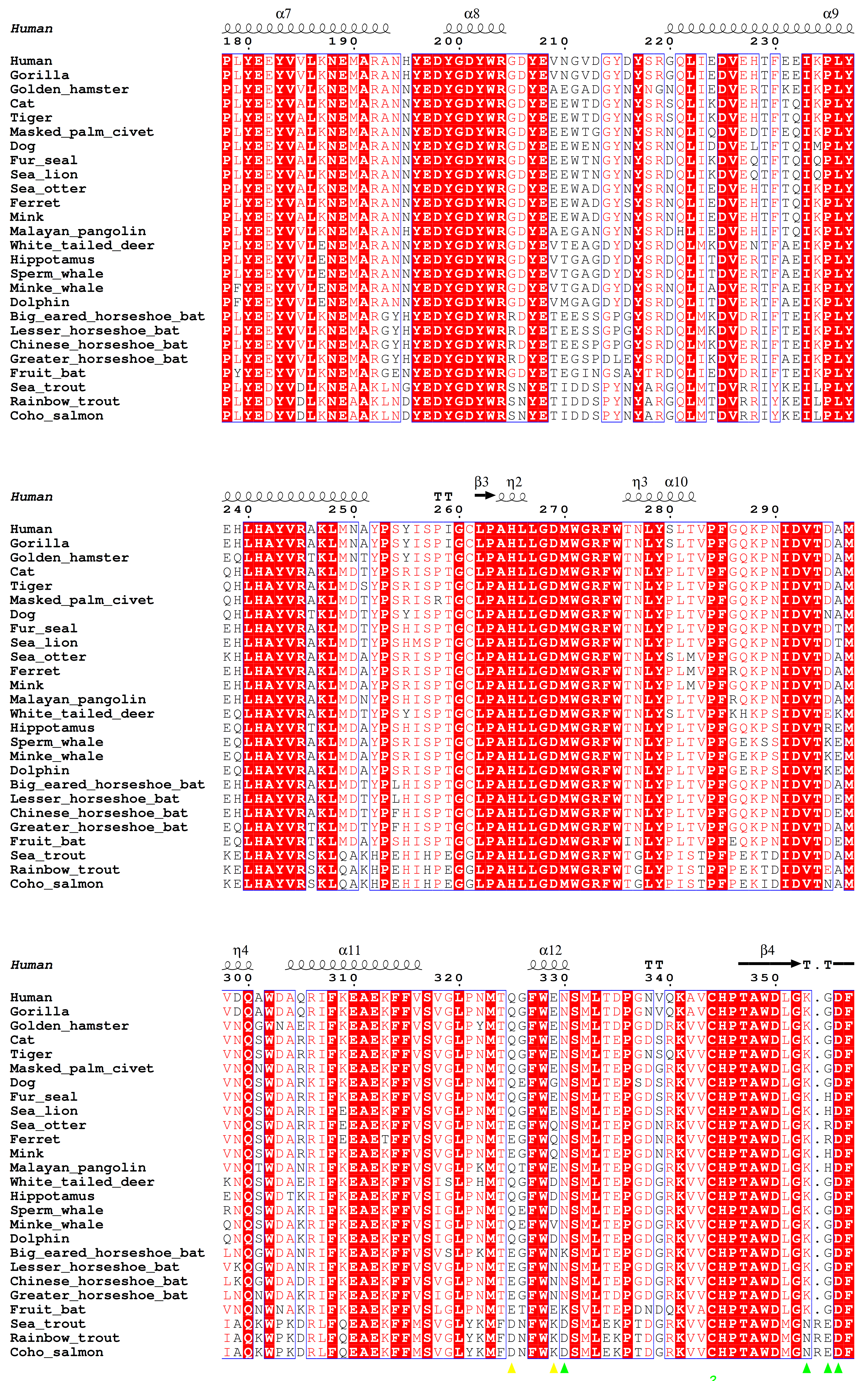


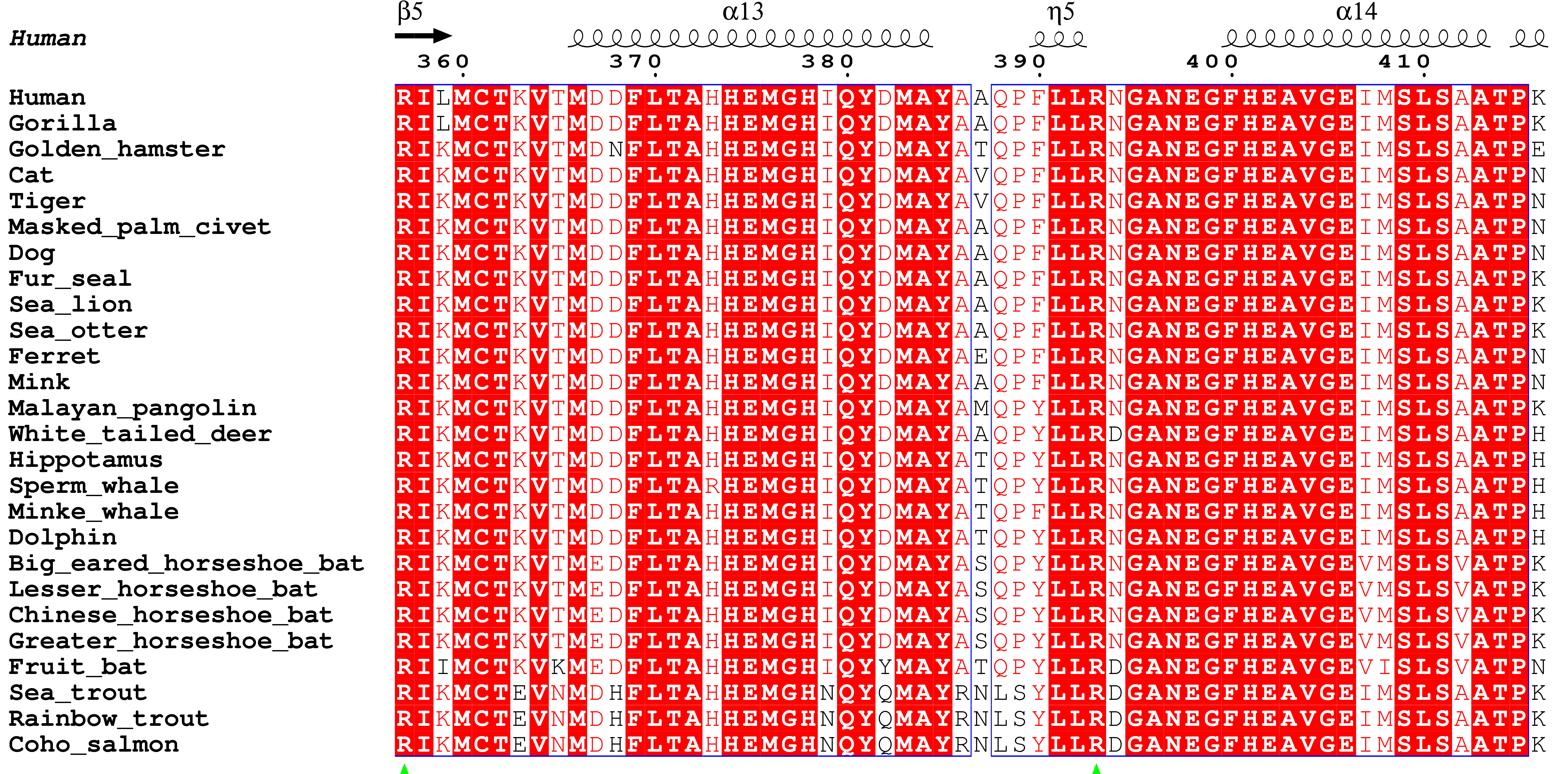


**Supplementary Figure 2.** Structure-based sequence alignment of 26 ACE2 orthologs, related to Supplementary Figure 1. Coils indicate α helices, and black arrows indicate β strands. Conserved residues are highlighted in red. Residues highlighted in blue boxes are highly (80%) conserved, with consensus amino acids in red. Green triangle indicates contacts with both SARS-CoV-2 RBD and SARS-CoV RBD, blue triangle indicates contacts with SARS-CoV-2 RBD alone, and yellow triangle indicates contacts with SARS-CoV RBD alone. The sequence alignment was generated with ClustalX and ESPript.


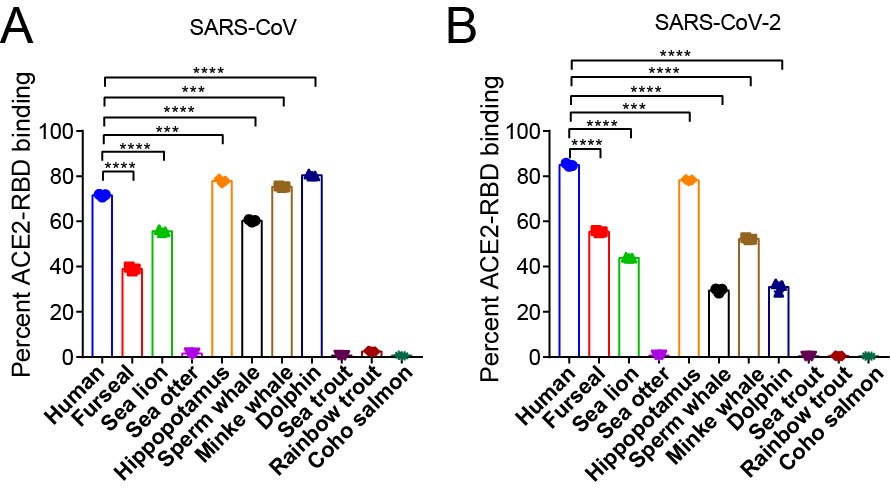


**Supplementary Figure 3.** Percentage of ACE2-positive cells among total EGFP-positive cells, related to Figure 1. The percentage of SARS-CoV RBD- or SARS-CoV-2 RBD-positive cells (A and B) in BHK-21 cells with different ACE2 orthologs. Data represent the results of three replicates, and error bars show the SD of each measurement. P values were analyzed using the student’s t test (** P<0.01; *** P<0.001, **** P<0.0001).


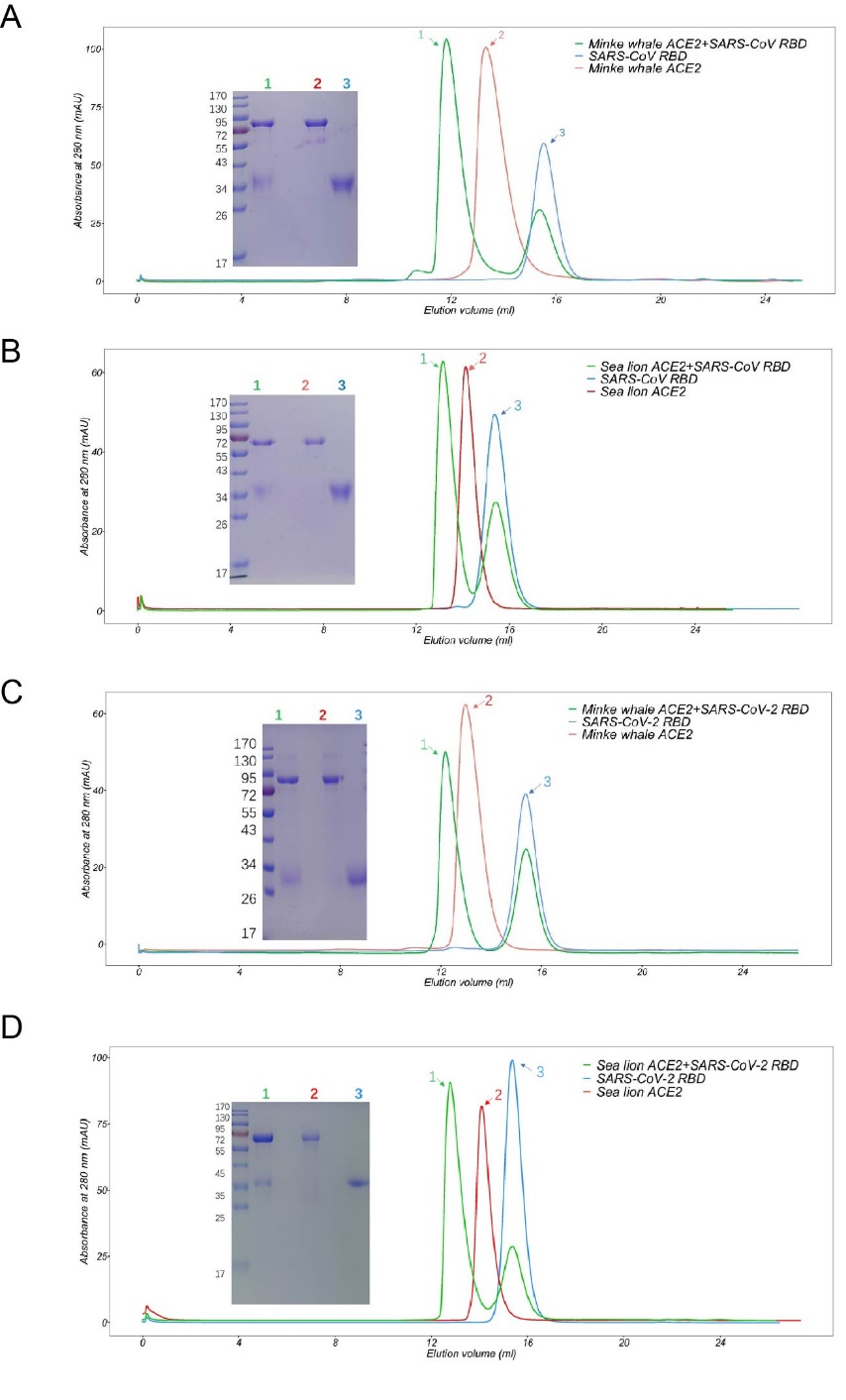


**Supplementary Figure 4.** Gel filtration profiles of ACE2, RBD and the ACE2/RBD complex. (A) MW-ACE2, SARS-CoV RBD and the complex. (B) SL-ACE2, SARS-CoV RBD and the complex. (C) MW-ACE2, SARS-CoV-2 RBD and the complex. (D) SL-ACE2, SARS-CoV-2 RBD and the complex. The separation profiles of each pooled sample on SDS-PAGE are shown in reducing conditions (+DTT).

**
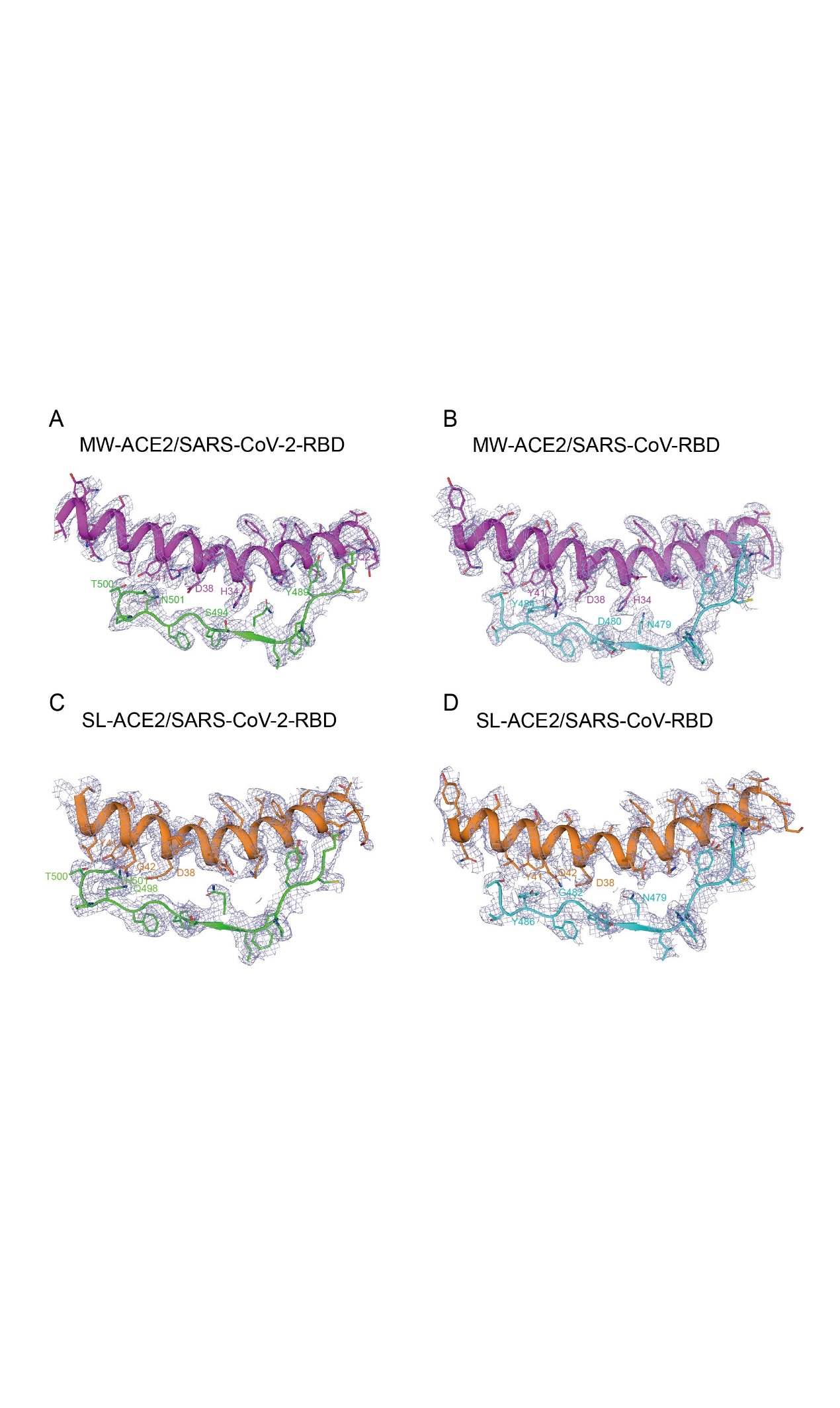
**

**Supplementary Figure 5.** Representative densities and atomic models of cryo-EM MW-ACE2/SARS-CoV-2 RBD (A), MW-ACE2/SARS-CoV RBD (B), SL-ACE2/ SARS-CoV-2 RBD (C) and SL-ACE2/SARS-CoV RBD (D). MW-ACE2, SL-ACE2, SARS-CoV-2 RBD and SARS-CoV RBD were shown as purple, orange, green and blue, respectively.

**
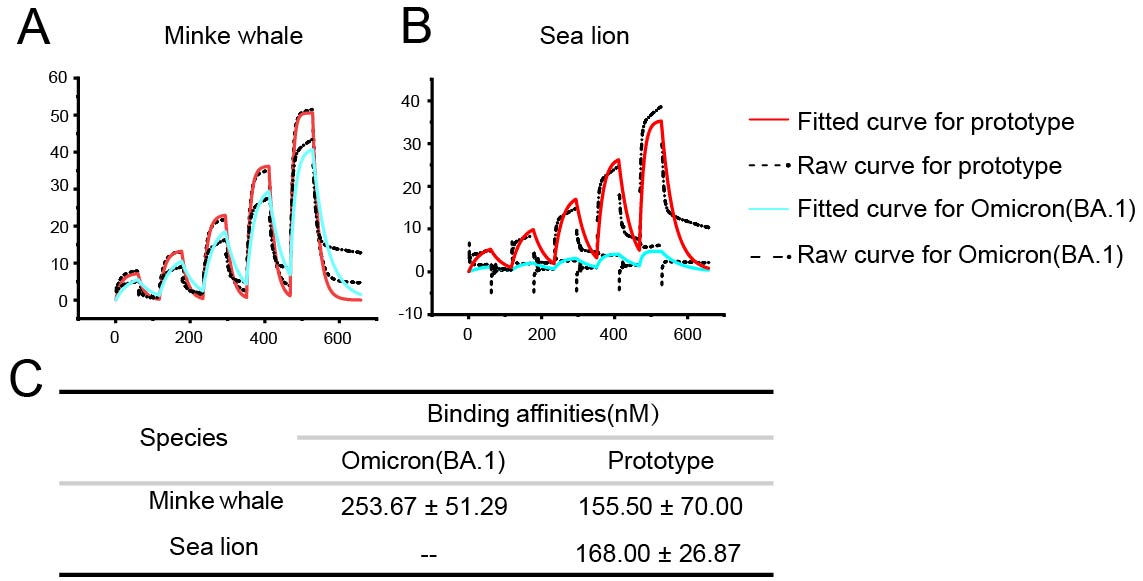
**

**Supplementary Figure 6.** Binding abilities of WM-ACE2 (A) and SL-ACE2 (B) with SARS-CoV-2 prototype and Omicron RBD. The binding affinities were shown in the table(C).


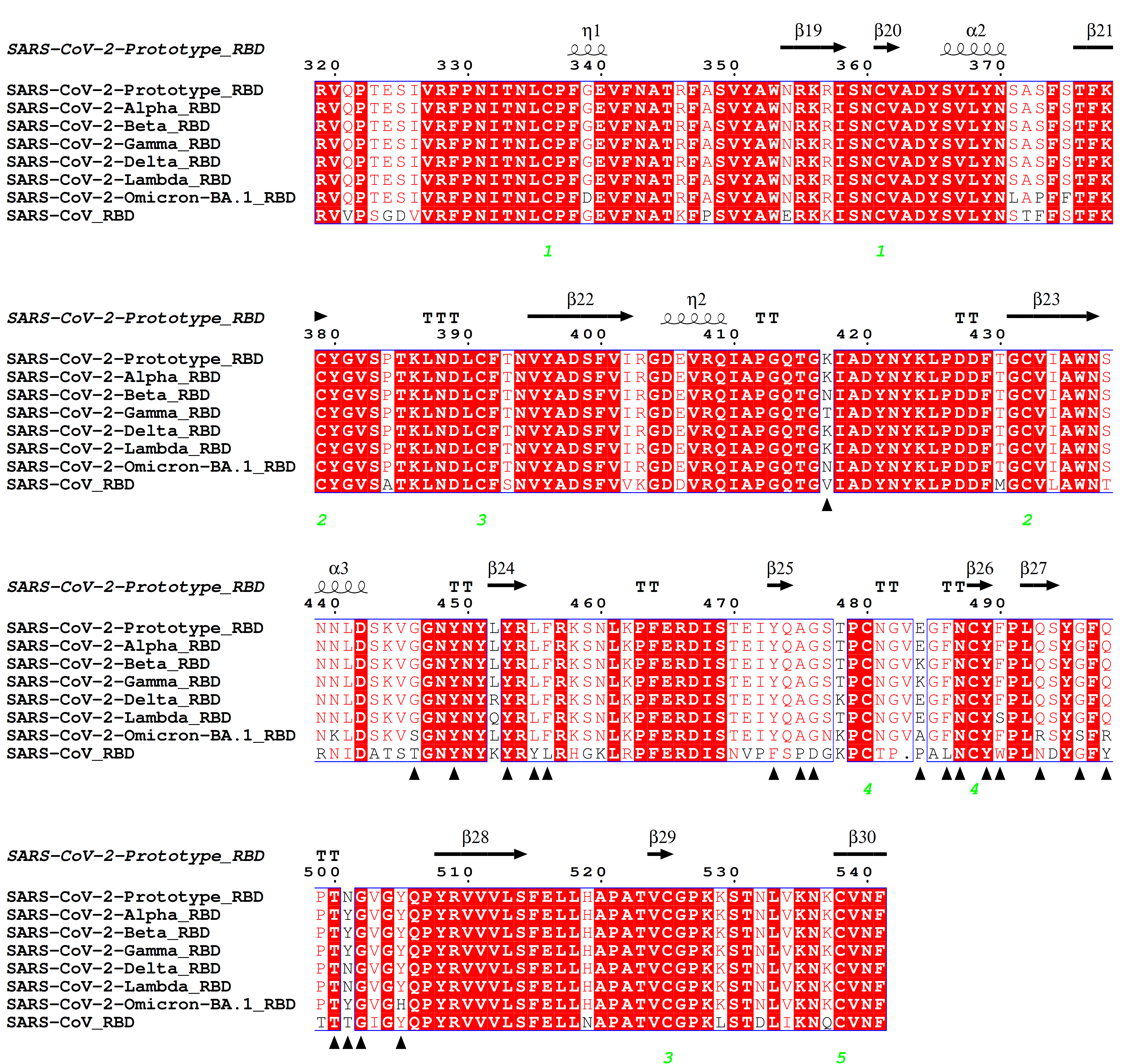


**Supplementary Figure 7.** Structure-based sequence alignment of RBDs of SARS-CoV, SARS-CoV-2 prototype and variants. Coils indicate α helices, and black arrows indicate β strands. Conserved residues are highlighted in red. Residues highlighted in blue boxes are highly (80%) conserved, with consensus amino acids in red. Interacting residues of prototype SARS-CoV-2 RBD to hACE2 are labeled with black triangles. The alignment is performed by T-COFFEE and visualized by ESPript 3.0.


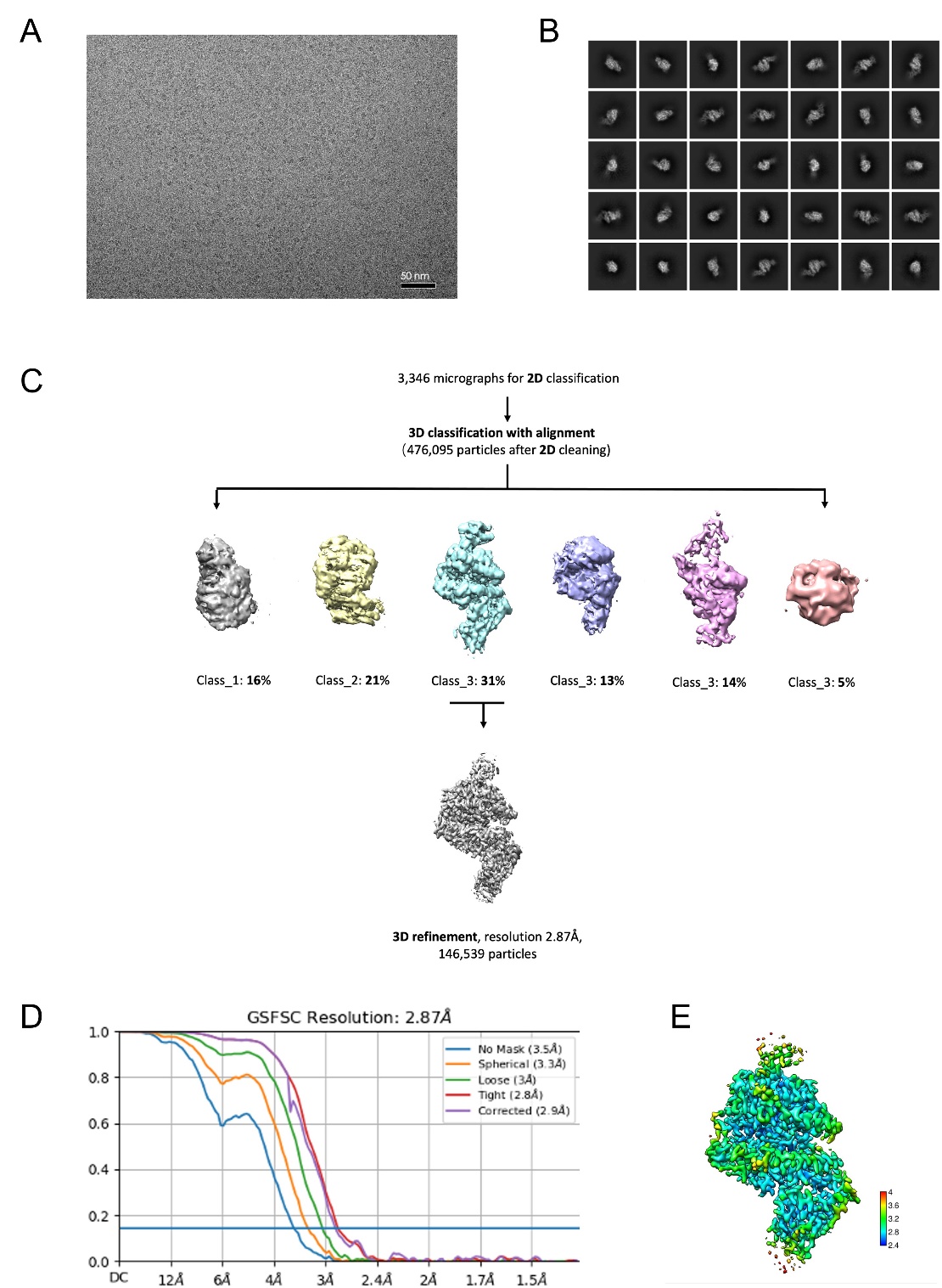


**Supplementary Figure 8.** Cryo-EM analysis of the MW-ACE2/SARS-CoV RBD complex. (A) A representative micrograph of the MW-ACE2/SARS-CoV RBD complex. The scale bar represents 50 nm. (B) Gallery of 2D class average images of the complex. (C) A brief workflow of cryo-EM image processing and reconstruction. (D) FSC curves for the reconstruction. (E) Local resolution distribution for the final density map.


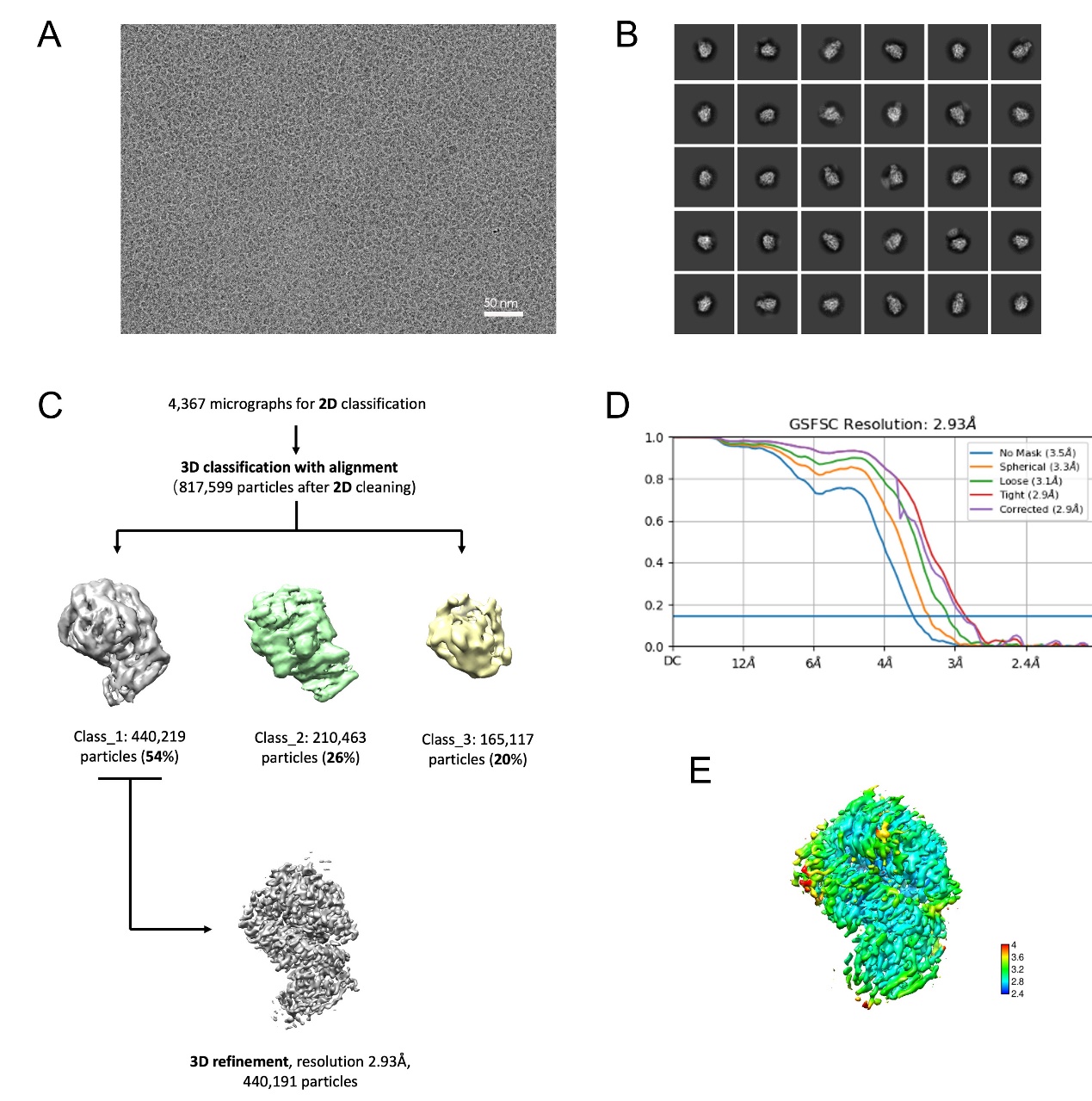


**Supplementary Figure 9.** Cryo-EM analysis of the MW-ACE2/SARS-CoV-2 RBD complex. (A) A representative micrograph of the MW-ACE2/SARS-CoV-2 RBD complex. The scale bar represents 50 nm. (B) Gallery of 2D class average images of the complex. (C) A brief workflow of cryo-EM image processing and reconstruction. (D) FSC curves for the reconstruction. (E) Local resolution distribution for the final density map.


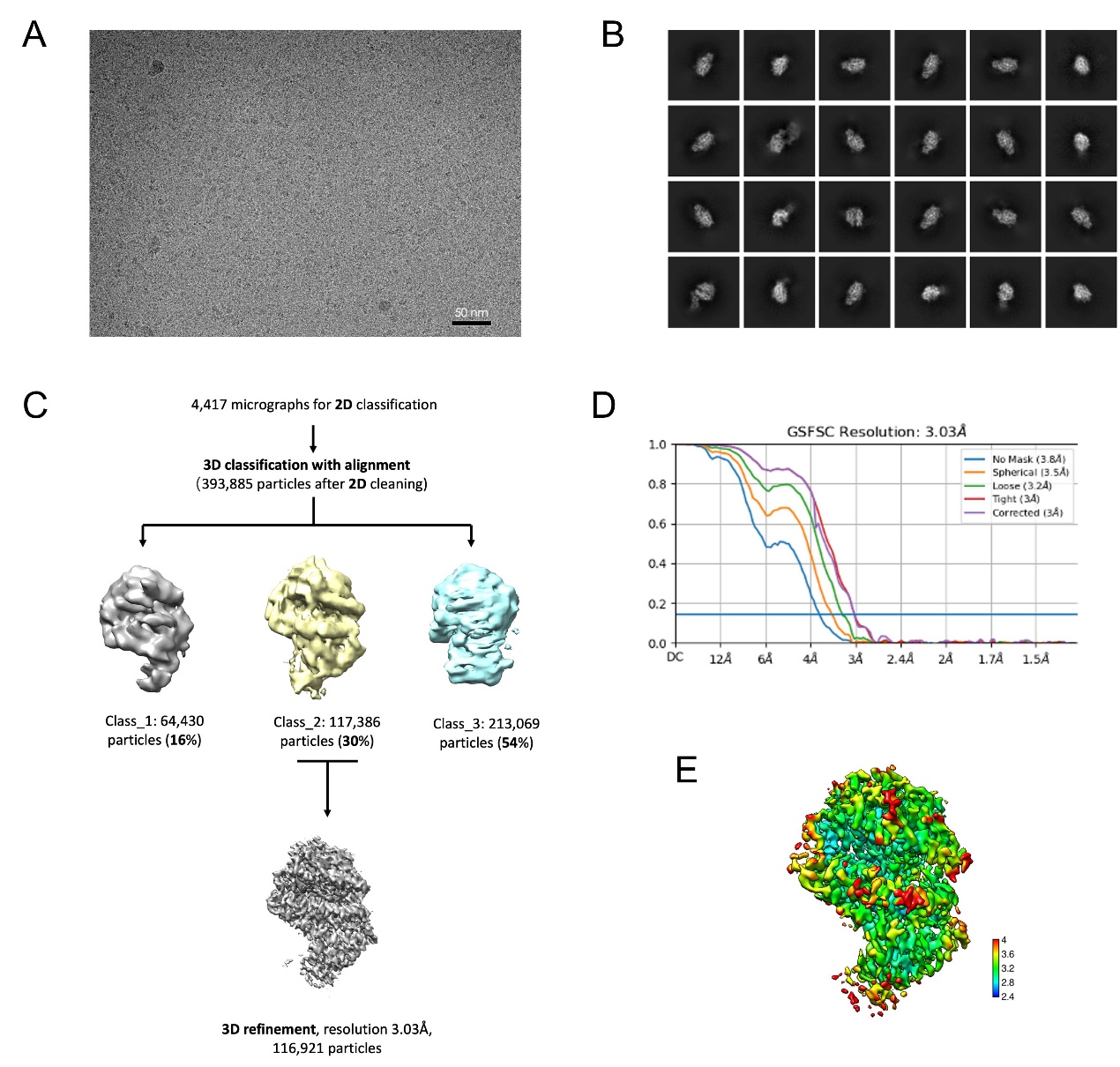


**Supplementary Figure 10.** Cryo-EM analysis of the SL-ACE2/SARS-CoV RBD complex. (A) A representative micrograph of the SL-ACE2/SARS-CoV RBD complex. The scale bar represents 50 nm. (B) Gallery of 2D class average images of the complex. (C) A brief workflow of cryo-EM image processing and reconstruction. (D) FSC curves for the reconstruction. (E) Local resolution distribution for the final density map.


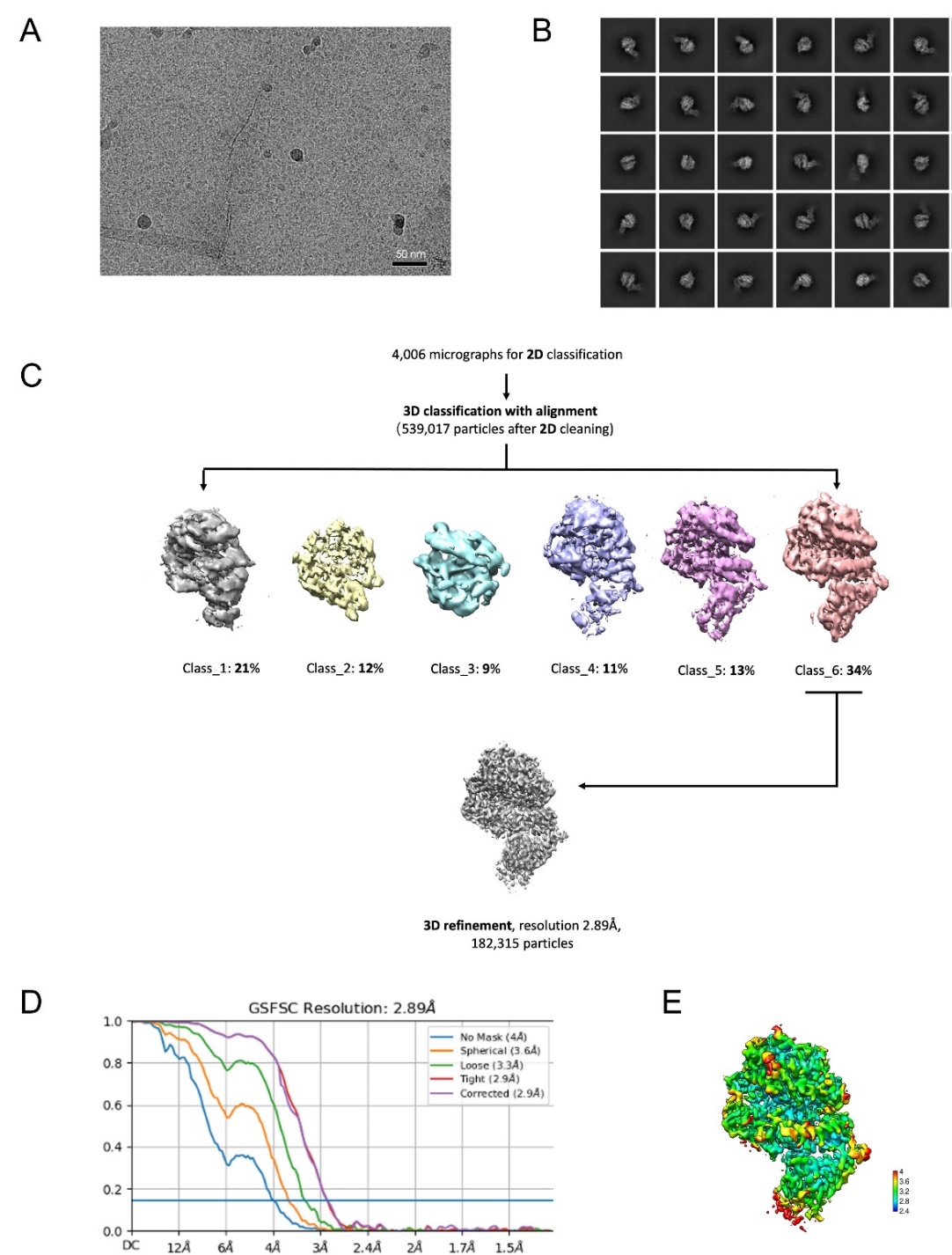


**Supplementary Figure 11.** Cryo-EM analysis of the SL-ACE2/SARS-CoV-2 RBD complex. (A) A representative micrograph of the SL-ACE2/SARS-CoV-2 RBD complex. The scale bar represents 50 nm. (B) Gallery of 2D class average images of the complex. (C) A brief workflow of cryo-EM image processing and reconstruction. (D) FSC curves for the reconstruction. (E) Local resolution distribution for the final density map.

**Supplementary Table 1.** Cryo-EM data processing and refinement statistics.

|  | MW-ACE2-SARS-CoV RBD | MW-ACE2-SARS-CoV-2 RBD | SL-ACE2-SARS-CoV RBD | SL-ACE2-SARS-CoV-2 RBD |
| --- | --- | --- | --- | --- |
| **Data collection and processing** |  |  |  |  |
| Microscope | Titan Krios G3i | Titan Krios G3i | Titan Krios G3i | Titan Krios G3i |
| Magnification | 105k | 105k | 105k | 105k |
| Voltage (kV) | 300 | 300 | 300 | 300 |
| Electron exposure (e^-^/Å^2^) | 50 | 50 | 50 | 50 |
| Defocus range (μm) | -1.0 ~ -2.0 | -1.0 ~ -2.0 | -1.0 ~ -2.0 | -1.0 ~ -2.0 |
| Pixel size (Å) | 0.669 | 0.669 | 0.669 | 0.669 |
| Symmetry imposed | C1 | C1 | C1 | C1 |
| Final particle images (no.) | 146539 | 440191 | 116921 | 182315 |
| Map resolution (Å) | 2.87 | 2.93 | 3.03 | 2.89 |
| FSC threshold | 0.143 | 0.143 | 0.143 | 0.143 |
| **Refinement** |  |  |  |  |
| Initial model used (PDB code) | 6LZG | 6LZG | 6LZG | 6LZG |
| Model resolution range (Å) | Up to 2.5 | Up to 2.5 | Up to 2.5 | Up to 2.5 |
| Map sharpening B factor (Å^2^) | 93.6 | 122.4 | 97.3 | 86.2 |
| **Map correlation coefficient** |  |  |  |  |
| Whole unit cell | 0.79 | 0.78 | 0.68 | 0.79 |
| Around atoms | 0.8 | 0.78 | 0.69 | 0.8 |
| **Model composition** |  |  |  |  |
| Non-hydrogen atoms | 7170 | 6431 | 6413 | 6457 |
| Protein residues | 879 | 791 | 787 | 793 |
| Nucleotide residues | - | - | - | - |
| **B-factor** |  |  |  |  |
| Protein | 157 | 159 | 157 | 147 |
| Nucleic acid | - | - | - | - |
| Ligand/ion | 30 | 30 | 30 | 87 |
| **R.m.s.deviations** |  |  |  |  |
| Bond length (Å) | 0.003 | 0.002 | 0.003 | 0.002 |
| Bond angles (°) | 0.439 | 0.446 | 0.53 | 0.44 |
| **Validation** |  |  |  |  |
| MolProbity score | 1.92 | 1.26 | 2.17 | 1.26 |
| Clashscore | 6.14 | 4.86 | 10.41 | 5.01 |
| Poor rotamers (%) | 3.86 | 1.01 | 4.6 | 0.72 |
| **Ramachandran analysis** |  |  |  |  |
| Most favored (%) | 97.26 | 98.22 | 97.32 | 98.10 |
| Allowed (%) | 2.74 | 1.78 | 2.68 | 1.90 |
| Disallowed (%) | 0.00 | 0.00 | 0.00 | 0.00 |

**Supplementary Table 2.** Comparison of amino acid residues of MW-ACE2, SL-ACE2 and hACE2 interacting with SARS-CoV-2 RBD

| MW-ACE2/SL-ACE2/hACE2 | SARS-CoV-2 RBD (MW-ACE2) | SARS-CoV-2 RBD (SL-ACE2) | SARS-CoV-2 RBD (hACE2) |
| --- | --- | --- | --- |
| Q18/R18/Q18 |  | S477 (4) |  |
| S19 | A475 (3), S477 (1) | A475 (2), G476 (1) | A475 (3, **1**), G476 (4) |
| Q24/ L24/Q24 | A475 (4), G476 (5),  N487 (10, **1**) | A475 (4), G476 (2), N487 (5), Y489 (1) | A475 (4), G476 (5),  N487(15, **1**) |
| T27 | F456 (10), Y473 (2),  A475 (2), Y489 (2) | F456 (9), Y473 (1), A475 (2), Y489 (9) | F456 (5), Y473 (1),  A475 (2), Y489 (7) |
| F28 | Y489 (11) | Y489 (8) | Y489 (7) |
| Q30/E30/D30 | K417 (6), L455 (2), F456 (5) | K417 (5), L455 (3), F456 (3) | K417 (4, **1**), L455 (2),  F456 (4) |
| K31 | L455 (2), F456 (10),  Y489 (12), Q493 (6) | L455 (3), F456 (11), Y489 (12), | L455 (2), F456 (5),  E484 (1), Y489 (6),  F490 (2), Q493 (3) |
| H34/S34/H34 | Y453 (8), L455 (1),  Q493 (20), S494 (12, **1**), Y495 (1) | Y453 (2), L455 (4), Q493 (10) | Y453 (5, **1**), L455 (9),  Q493 (6) |
| E35 | Q493 (5) | Q493 (6) | Q493 (8, **1**) |
| E37 | D403 (1), Y505 (4) | Y505 (2) | Y505 (7) |
| D38/E38/D38 | Y449 (9, **1**), G496 (6), Q498 (1) | Y449 (11, **1**), G496 (6),  Q498 (6, **1)** | Y449 (9, **1**), G496 (5),  Q498 (1) |
| Y41 | Q498 (5), T500 (7, **1**), N501 (10) | Q498 (9), T500 (7,**1**),  N501 (10) | Q498 (8), T500 (7, **1**),  N501 (8) |
| R42/Q42/Q42 | G446 (1), Y449 (3), Q498 (2) | G446 (4), Y449 (4, **1**),  Q498 (3) | G446 (4, **1**), Y449 (4, **1**), Q498 (8, **1**) |
| L45 | Q498 (1), T500(3) | T500 (2) | Q498 (3), T500 (1) |
| I79/Q79/L79 | F486 (3) | F486 (6) | F486 (2) |
| T82/T82/M82 | F486 (5) |  | F486 (9) |
| Y83 | F486 (13), N487 (4, **1**), Y489 (1, **1**) | N487 (4, **1**), Y489 (1) | F486 (11), N487 (8, **1**),  Y489 (1) |
| N329/N330/N330 | T500 (2) | T500 (9) | T500 (8) |
| E325/G326/G326 | N501 (1), V503 (3), Q506 (3) |  |  |
| K352/K353/K353 | G496 (5, **1**), N501 (12), G502 (3, **1**), Y505 (28) | G496 (3), Q498 (1), N501 (12), G502 (6, **1**), Y505 (22) | G496 (7, **1**), N501 (11),  G502 (4, **1**), Y505 (28) |
| G353/H354/G354 | G502 (6), Y505 (5) | D405 (4, **1**), G502 (8),  G504 (1), Y505 (15) | G502 (7), Y505 (4) |
| D354/D355/D355 | T500 (9), N501 (1), G502 (1) | T500 (8), G502 (3) | T500 (8), G502 (1) |
| R356/R357/R357 | T500 (6) | T500 (5) | T500 (3) |
| R392/R393/R393 | Y505 (3) | Y505 (1) | Y505 (1) |
| Total | 307, **8** | 280, **7** | 288, **13** |

The numbers without underlines in parentheses of SARS-CoV-2 RBD (MW-ACE2), SARS-CoV-2 RBD (SL-ACE2) and SARS-CoV-2 RBD (hACE2) residues represent the number of vdw contacts between the indicated SARS-CoV-2 RBD residues with the MW-ACE2, SL-ACE2 or hACE2. Underlined numbers in bold suggest the number of potential H-bonds between the pairs of residues. vdw contacts were analyzed at a cutoff of 4.5 Å and H-bonds at a cutoff of 3.5 Å.

**Supplementary Table 3.** Comparison of amino acid residues of MW-ACE2, SL-ACE2 and hACE2 interacting with SARS-CoV RBD.

| MW-ACE2/SL-ACE2/hACE2 | SARS-CoV RBD (MW-ACE2) | SARS-CoV RBD (SL-ACE2) | SARS-CoV RBD (hACE2) |
| --- | --- | --- | --- |
| S19 | P462 (8), D463 (5) | D463 (5) | P462 (1) |
| E23/D23/E23 |  | P462 (1) |  |
| Q24/L24/Q24 | N473 (16), Y475 (1), P462 (2) | P462 (5), D463 (1)  N473 (7), Y475 (1) | N473 (6, **1**) |
| T27 | L443 (4), Y475 (11), P462 (2) | L443 (1), P462 (8)  Y475 (9) | L443 (3), Y475 (5) |
| F28 | Y475 (6) | Y475 (8) | Y475 (7) |
| Q30/E30/D30 | Y442 (7), L443 (1) | Y442 (16), L443 (4) | Y442 (2) |
| K31 | Y442 (7), Y475 (7) | Y475 (17) | Y442 (6), Y475 (6) |
| D33/N33/N33 |  | Y442 (1) |  |
| H34/S34/H34 | Y440 (9, **1**), N479 (19), D480 (8, **1**) | Y442 (8), N479 (5) | Y440 (5, **1**), Y442 (1), N479 (4) |
| E35 |  | N479 (1) |  |
| E37 | Y491 (5) | Y491 (5) | Y491 (4) |
| D38/E38/D38 | Y436 (11, **1**), Y484 (4) | Y436 (9, **1**), D480 (2)  Y481 (2), G482 (7, **1**), | Y436 (9, **2**), G482 (1), Y484 (1) |
| Y41 | Y484 (15), T486 (7, **1**), T487 (7) | Y484 (7), T486 (5, **1**), T487 (7) | Y484 (9), T486 (8, **1**), T487 (8) |
| R42/Q42/Q42 | T433 (1), Y436 (10, **1**), Y484 (6, **1**) | Y436 (6, **1**), Y484 (1) | Y436 (5, **1**), Y484 (4) |
| L45 | S432 (1), Y484 (2), T486 (1) | Y484 (7), T486 (1) | Y484 (2), T486 (1) |
| I79/Q79/L79 | L472 (1) | L472 (4) | L472 (2) |
| T82/T82/M82 | L472 (1) |  | L472 (4) |
| Y83 | N473 (6, **1**), Y475 (1) | N473 (7, **1**), Y475 (1) | N473 (8, **2**), Y475 (2) |
| T323/T324/T324 |  | I489 (4) |  |
| Q324/ Q325/Q325 | I489 (1) | I489 (6) | R426 (2), I489 (2) |
| E325/G326/G326 | R426 (2), T485 (1), T486 (2), T487 (5), G488 (1), Q492 (5, **1**) |  |  |
| V328/E329/E329 |  |  | R426 (6, **1**) |
| N329/N330/N330 | T486 (6, **1**) | R426 (1), T486 (14), T487 (1) | T486 (11, **1**) |
| K352/K353/K353 | G482 (4), Y484 (3), T487 (9), G488 (3, **1**), Y491 (26) | G482 (7), T487 (7),  G488 (3, **1**),  Y491 (29) | Y481 (1), G482 (3), Y484 (2), T487 (11), G488 (6, **1**),  Y491 (25) |
| G353/H354/G354 | G488 (6), Y491 (6) | D392 (2), G488 (8,**1**), Y491 (12), T487 (1) | G488 (7), Y491 (3) |
| D354/D355/D355 | T486 (8), T487 (1),  G488 (1) | T486 (9), T487 (4)  G488 (2) | T486 (8), T487 (3), G488 (4) |
| R356/R357/R357 | T486 (3) | T486 (7) | T486 (4) |
| R392/R393/R393 |  |  | Y491 (1) |
| Total | 285, **10** | 286,**7** | 213, **11** |

The numbers without underlining in parentheses of SARS-CoV RBD (MW-ACE2), SARS-CoV RBD (SL-ACE2) and SARS-CoV RBD (hACE2) residues represent the number of vdw contacts between the indicated SARS-CoV RBD residues and the MW-ACE2, SL-ACE2, or hACE2. Underlined numbers in bold suggest the number of potential H-bonds between the pairs of residues. vdw contact were analyzed at a cutoff of 4.5 Å and H-bonds at a cutoff of 3.5 Å.

**Supplementary Table 4.** The accession numbers of ACE2s.

| **Species of ACE2** | **Organism** | **Database** | **Code** |
| --- | --- | --- | --- |
| Human | *Homo sapiens* | NCBI | NP_001358344.1 |
| Gorilla | *Gorilla gorilla gorilla* | NCBI | XP_018874749.1 |
| Golden hamster | *Mesocricetus auratus* | NCBI | XP_005074266.1 |
| Cat | *Lynx canadensis* | NCBI | XP_030160839.1 |
| Tiger | *Panthera tigris* | NCBI | XP_007090142.2 |
| Masked palm civet | *Paguma larvata* | GenBank | AAX63775.1 |
| Dog | *Canis lupus familiaris* | NCBI | XP_005641049.1 |
| Fur seal | *Callorhinus ursinus* | NCBI | XP_025713397.1 |
| Sea lion | *Eumetopias jubatus* | NCBI | XP_027970822.1 |
| Sea otter | *Enhydra lutris kenyoni* | NCBI | XP_022374078.1 |
| Ferret | *Mustela putorius furo* | GenBank | BAE53380.1 |
| Mink | *Neovison vison* | GenBank | *QPL12211.1* |
| Malayan pangolin | *Manis javanica* | NCBI | XP_017505746.1 |
| White tailed deer | *Odocoileus virginianus* | NCBI | XP_020768965.1 |
| Hippopotamus | *Hippopotamus amphibius* | Genbank | NKPW01009838.1 |
| Sperm whale | *Physeter catodon* | NCBI | XP_023971279.1 |
| Minke whale | *Balaenoptera acutorostrata scammoni* | NCBI | XP_028020351.1 |
| Dolphin | *Phocoena sinus* | NCBI | XP_032476004.1 |
| Big-eared horseshoe bat | *Rhinolophus macrotis* | GenBank | ADN93471.1 |
| Lesser horseshoe bat | *Rhinolophus pusillus* | GenBank | ADN93477.1 |
| Chinese horseshoe bat | *Rhinolophus sinicus* | NCBI | AGZ48803.1 |
| Greater horseshoe bat | *Rhinolophus ferrumequinum* | NCBI | BAH02663.1 |
| Fulvous fruit bat | *Rousettus leschenaultii* | GenBank | ADJ19219.1 |
| Sea trout | *Salmo trutta* | NCBI | XP_029603502.1 |
| Rainbow trout | *Oncorhynchus mykiss* | NCBI | XP_021433278.1 |
| Coho salmon | *Oncorhynchus kisutch* | NCBI | XP_031677944.1 |

**REFERENCES**

1. Wu, L, Chen, Q, Liu, K*, et al.* Broad host range of SARS-CoV-2 and the molecular basis for SARS-CoV-2 binding to cat ACE2. *Cell Discov* 2020; **6**: 68.

2. Niu, S, Wang, J, Bai, B*, et al.* Molecular basis of cross-species ACE2 interactions with SARS-CoV-2-like viruses of pangolin origin. *EMBO J* 2022; **41**: e109962.

3. Muik, A, Wallisch, AK, Sanger, B*, et al.* Neutralization of SARS-CoV-2 lineage B.1.1.7 pseudovirus by BNT162b2 vaccine-elicited human sera. *Science* 2021; **371**: 1152-3.
